# Human breastmilk memory T cells throughout lactation manifest activated tissue-oriented profile with prominent regulation

**DOI:** 10.1172/jci.insight.181788

**Published:** 2024-09-03

**Authors:** Elise S. Saager, Arthur H. van Stigt, Butstabong Lerkvaleekul, Lisanne Lutter, Anneke H. Hellinga, M. Marlot van der Wal, Louis J. Bont, Jeanette H.W. Leusen, Belinda van’t Land, Femke van Wijk, the Protection against Respiratory tract infections through human Milk Analysis (PRIMA) group

**Affiliations:** 1 Center for Translational Immunology, University Medical Centre Utrecht, Utrecht, Netherlands.; 2 Division of Rheumatology, Department of Pediatrics, Faculty of Medicine Ramathibodi Hospital, Mahidol University, Bangkok, Thailand.; 3 Department of Pathology, Amsterdam University Medical Centre, Amsterdam, Netherlands.; 4 Department of Paediatric Immunology and Infectious Diseases, Wilhelmina Children’s Hospital/University Medical Center Utrecht, Utrecht, Netherlands.; 5 ReSViNET foundation, Zeist, Netherlands.; 6 CoE Immunology, Danone Global Research & Innovation Center, Utrecht, Netherlands.; 7 Supplemental Acknowledgments.

**Keywords:** Immunology, T cells

## Abstract

Breastfeeding provides important immunological benefits to the neonate, but how the different immunoactive components in breastmilk contribute to immunity remains poorly understood. Here, we characterized human breastmilk T cells using single-cell RNA-Seq and flow cytometry. Breastmilk contained predominantly memory T cells, with expression of immune signaling genes, high proliferation, and an effector Th1/cytotoxic profile with high cytokine production capacities. Elevated activation was balanced by an enriched Treg population and immune regulatory markers in conventional memory T cells. Gene and surface expression of tissue-residency markers indicate that breastmilk T cells represented tissue-adapted rather than circulatory T cells. In addition, breastmilk T cells had a broad homing profile and higher activation markers in these migratory subsets. The partly overlapping transcriptome profile between breastmilk and breast tissue T cells, particularly cytotoxic T cells, might support a role in local immune defense in the mammary gland. However, unique features of breastmilk, such as Tregs, might imply an additional role in neonatal immune support. We found some correlations between the breastmilk T cell profile and clinical parameters, most notably with maternal and household factors. Together, our data suggest that breastmilk contains an adapted T cell population that exerts their function in specific tissue sites.

## Abstract 2

Breastmilk T cells have a specialized profile, balancing between strong effector functions and regulation. Tissue markers indicate a location-specific function, potentially in the breast tissue.

## Introduction

The developing neonatal immune systems differs substantially from the adult immune system, with a small memory T cell compartment and differences in the extent and direction of inflammatory responses (reviewed in ref. 1). Although potentially functional in tolerance induction and prevention of immune-mediated damage (2), these differences might leave neonates susceptible to infections (reviewed in ref. 3). Breastmilk plays an important role in providing protection and support during this period of immune development early in life. Breastfed infants have lower infection and hospital admissions rates for common pathogens like RSV (4–6), effects that can continue for years after cessation of breastfeeding (4). In addition, breastmilk might affect tolerogenic immune development, with some studies reporting a decreased incidence of allergic diseases in exclusively breastfed infants, although conflicting results have been published (7–9).

Breastmilk contains a range of immunoactive components, including antibodies, lactoferrin, cytokines, fatty acids, oligosaccharides, microbiota, extracellular vesicles, miRNAs, and vital immune cells (10–15). The cellular immune fraction of human breastmilk contains monocytes/macrophages, neutrophils, and lymphocytes (16–20); T cells predominate in lymphocytes (20–23). The leukocyte compartment changes with lactation stage, with highest total leukocyte numbers in colostrum (<1 week postpartum: 1 × 10^5^ cells/mL versus 1 × 10^3^/mL to 1 × 10^4^/mL in mature milk) (17, 19, 20), including 3-fold higher T cell numbers in colostrum (6 ×10^3^/mL) versus mature milk (~2.10^3^/mL) (20). Increased T cell numbers were observed in breastmilk during maternal infections, especially local breast inflammation (mastitis) (17, 18). A distinct function of the breastmilk T cell population is further supported by enrichment of antigen-specific T cells in breastmilk (24–27) and predominant expression of memory and activation markers (23, 25, 28).

Animal studies suggest an active role for breastmilk T cells in neonatal immune defense. In mice, breastmilk CD8^+^ T cells could pass the neonatal intestinal barrier and successfully engraft in the neonatal Peyer’s patches and intestinal mucosa (29). Engrafted maternal CD8^+^ T cells produced high levels of inflammatory cytokines and granzyme B, indicating support of the underdeveloped Th1 response in neonates. Both in mice and rats, parasite-specific CD4^+^ breastmilk T cells could also engraft in neonatal lung and intestinal tissues and actively contributed to pathogen-specific immunity (30, 31).

In humans, proof of neonatal immune support by breastmilk T cells is lacking, but the association of maternal-infant chimerism with breastfeeding (32, 33) might indicate absorption of maternal T cells. Currently, phenotypic information on human breastmilk T cells is limited and poorly integrated, with studies profiling only a handful of markers at the same time and not correcting for the predominant memory skewing compared with peripheral blood. Here, we combined single-cell RNA-Seq (scRNA-Seq) and comprehensive flow cytometry in order to obtain an overview of the breastmilk T cell compartment and its potential functionality in humans. We used longitudinal breastmilk samples that were collected as part of the prospective PRIMA cohort, which couples clinical data with longitudinal breastmilk sampling in mother-child pairs (34).

## Results

### General composition of the breastmilk T cell compartment throughout lactation.

**Figure 1:**
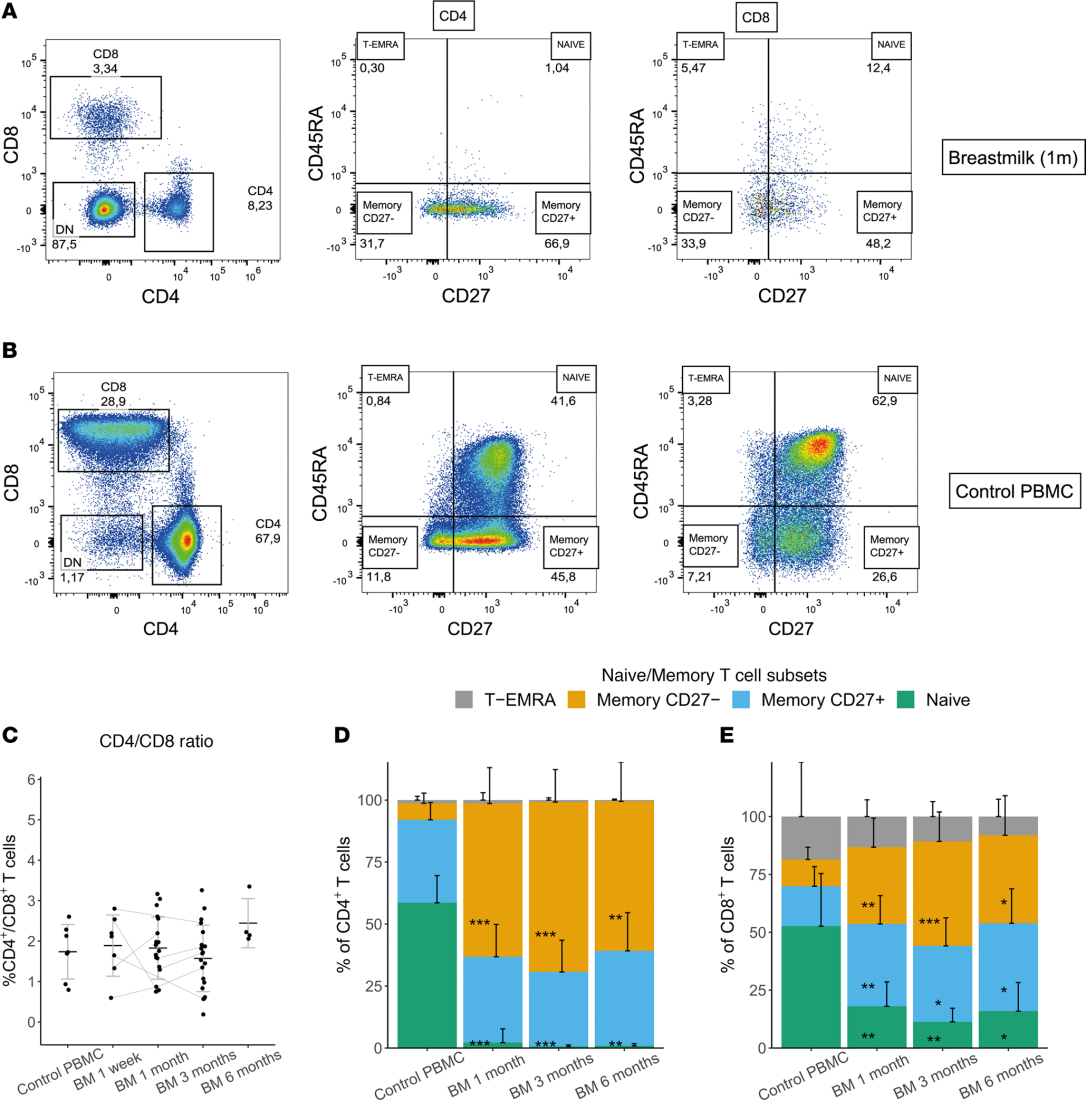
General breastmilk T cell composition. (**A** and **B**) Representative flow cytometry plots of the CD4^+^ and CD8^+^ naive/memory composition based on CD45RA and CD27 staining in breastmilk- and PBMC-derived T cells. (**C**) Ratio of CD4^+^ to CD8^+^ T cells, excluding TCRγδ^+^ T cells and MAIT cells (PBMC *n* = 7, breastmilk [BM] 1 week *n* = 7, BM 1 month *n* = 19, BM 3 months *n* = 20, BM 6 months *n* = 4). (**D** and **E**) Distribution of naive (CD45RA^+^CD27^+^), memory (CD45RA^–^, split up in CD27^–^ and CD27^+^) and T-EMRA (CD45RA^+^CD27^–^) T cell subsets expressed as the percentage of CD4^+^ (**D**) and CD8^+^ (**E**) T cells, with pairwise comparisons between BM time points and PBMC (PBMC *n* = 8, BM 1 month *n* = 20, BM 3 months *n* = 16, BM 6 months *n* = 12). Data represent mean ± SD. *P < 0.05, **P < 0.01, ***P < 0.001 using the Kruskal-Wallis test followed by Dunn’s test for multiple comparisons. For **D** and **E**, significance only denotes differences between BM timepoints compared with control PBMC. Transparent lines connect data points of different time points postpartum from the same breastmilk donor. MAIT, Musocal-Associated Invariant T cells (TCRVα7.2^+^CD161^+^); TCR, T cell receptor. FACS data of T cells in breastmilk of 1 week and 1, 3, and 6 months postpartum compared with PBMC of age-matched female control donors.

Live T cells were present in human breastmilk samples in a comparable proportion across all time points postpartum (Figure 1, A and B, and Supplemental Figure 1A; supplemental material available online with this article; https://doi.org/10.1172/jci.insight.181788DS1). Across all of our time points, breastmilk contained, on average, more CD4^+^ than CD8^+^ conventional T cells (TCRγδ^–^ and TCRVα7.2^–^), comparable with the ratio in PBMC (Figure 1C). Mucosal-associated invariant T cells (MAIT) (TCRVα7.2^+^CD161^+^) were numerous in a few breastmilk samples but not significantly different from PBMC (Supplemental Figure 1B). TCRγδ^+^ T cells were less frequent in breastmilk (Supplemental Figure 1C). The percentage of double-negative (DN) (CD4^–^CD8^–^) T cells was highly variable (range, 1%–99%; mean, 42%; Supplemental Figure 1D), and samples with many DN T cells (>50% of viable CD3^+^) were not enriched for either MAIT or TCRγδ^+^ T cells.

The majority of CD4^+^ and CD8^+^ breastmilk T cells had low expression of CD45RA, indicating an enriched memory population (Figure 1, D and E). For CD4^+^ breastmilk T cells, we observed only a significant increase in CD45RA^–^CD27^–^ memory T cells, whereas in CD8^+^ T cells, both the CD45RA^–^CD27^–^ and CD45RA^–^CD27^+^ population were significantly increased compared with PBMC (Figures 1, D and E and Supplemental Figure 2, A, B, E, and F). Although not assessed in combination with CD45RA, lower expression of CCR7 in CD4^+^ and CD8^+^ T cells further indicated a prominent effector memory T cell population in breastmilk (Supplemental Figure 2J). In contrast to CD4^+^, the CD8^+^ population also included CD45RA^+^CD27^–^ terminally differentiated effector memory cells reexpressing CD45RA (T-EMRA), comparable with PBMC, and maintained a small population of CD45RA^+^CD27^+^ naive T cells (Figure 1, D and E, and Supplemental Figure 2, C, D, G, and H). Although in total reduced compared with PBMC (Figure 1E), a larger fraction of these naive CD8^+^ T cells were CD31^–^ (Supplemental Figure 2I), possibly indicating increased differentiation (35) or contamination with T-EMRA.

### scRNA-Seq of breastmilk T cells.

**Figure 2:**
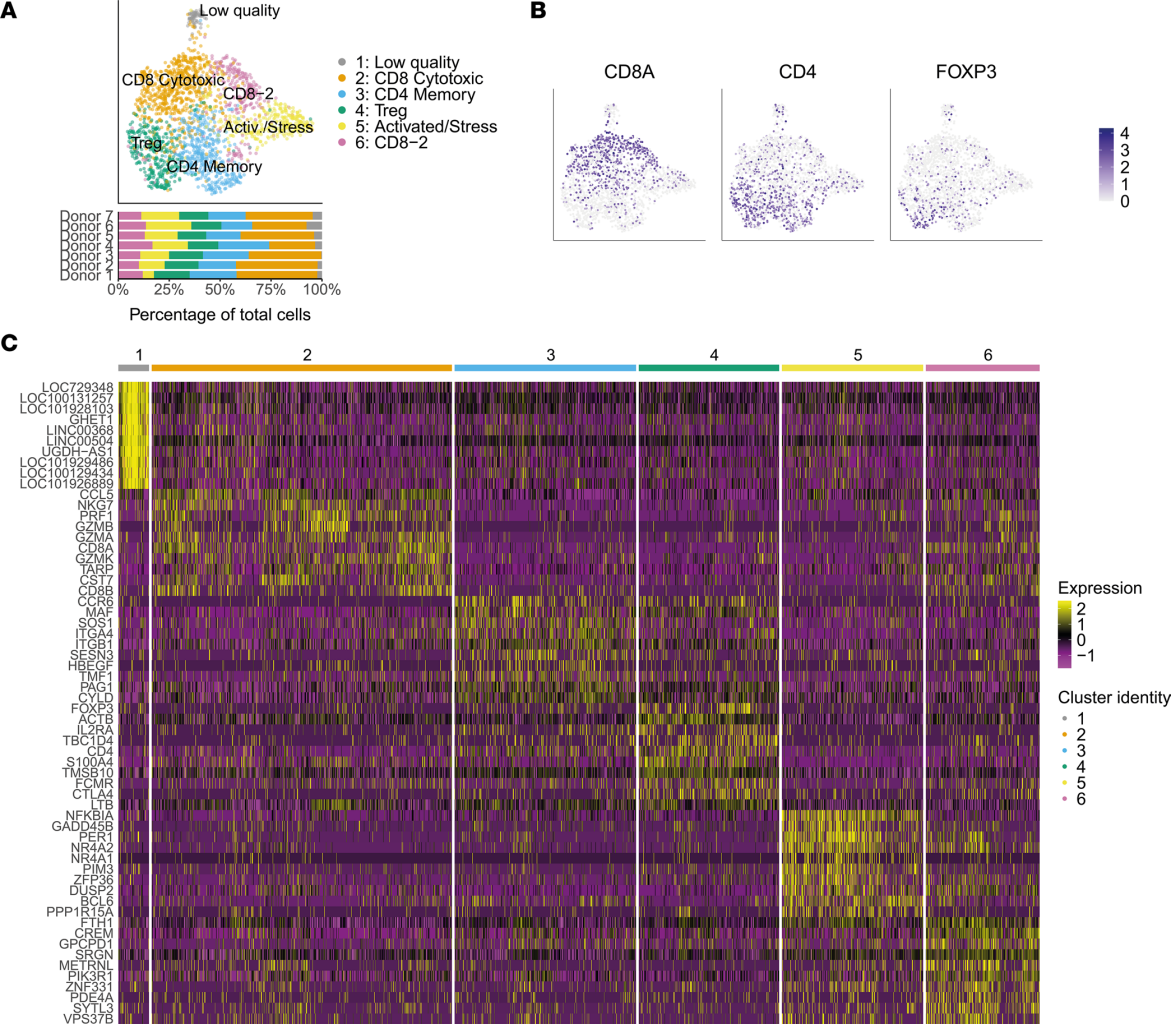
Breastmilk T cell populations identified by single-cell RNA-seq. T cell populations present in breastmilk at 1 month postpartum (*n* = 7). (**A**) UMAP showing cluster annotation and cluster proportions per breastmilk donor. (**B**) UMAP gene expression (log-normalized) projections for CD8A, CD4, and FOXP3. (**C**) Heatmap showing expression (log-normalized) of the top 10 differentially expressed genes (DEG, MAST test) per cluster.

To get a comprehensive and unbiased overview of the breastmilk T cell population, we performed scRNA-Seq on live CD3^+^ T cells from 1 month postpartum breastmilk (Supplemental Figure 3, D–F). We identified 6 distinct populations (Figure 2A), with *CD8A* and *CD8B* expression in clusters 2 and 6 and *CD4* expression in clusters 3 and 4, of which cluster 4 coexpressed *FOXP3*, indicating Tregs (Figure 2, B and C). Cluster 1 was very distinct, with high log fold changes in the differentially expressed genes (DEG; Figure 2C), including many long noncoding RNAs, possibly indicating either contamination or low-quality cells (Supplemental Figure 3, D–F). More than half of the cells in clusters 5 and 1 expressed neither *CD4* nor *CD8A/B*. Using the index sort surface expression data (Supplemental Figure 3, A–C), we confirmed that the highest fraction of DN cells was present in cluster 5 (24%).

### Breastmilk T cells demonstrate an activated/effector profile with cytotoxicity and high cytokine production capacity.

**Figure 3:**
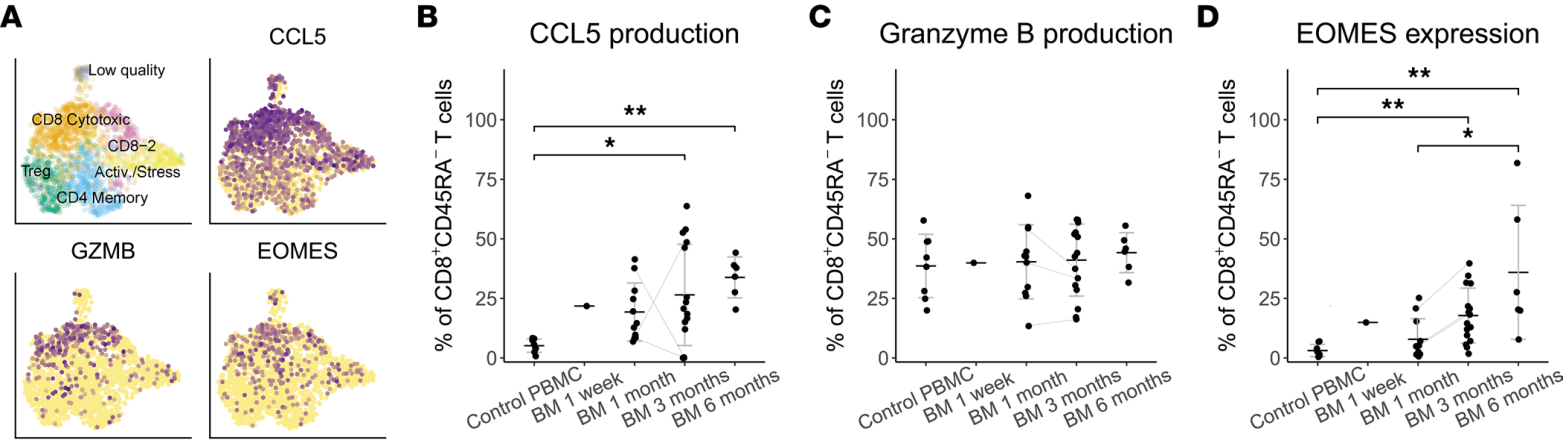
Functional profile of breastmilk cytotoxic T cells. (**A**) UMAP showing clustering and gene expression (log-normalized) projections for the cluster 2 characteristic genes CCL5, granzyme B, and EOMES in 1 month postpartum breastmilk (single-cell RNA-Seq, *n* = 7). (**B**–**D**) Frequency of CCL5^+^ (**B**), granzyme B^+^ (**C**), and EOMES^+^ (**D**) cells as the percentage of CD8^+^CD45RA^–^ T cells in breastmilk of 1 week and 1, 3, and 6 months postpartum compared with PBMC of age-matched female control donors (FACS) (PBMC *n* = 8, BM 1 week *n* = 1, BM 1 month *n* = 11, BM 3 months *n* = 16, BM 6 months *n* = 6). *P < 0.05,**P < 0.01, with pairwise comparisons among PBMC and BM across different time points using the Kruskal-Wallis test followed by Dunn’s test for multiple comparisons. Data represent mean ± SD. Gray lines connect data points of different time points from the same breastmilk donor. BM, breastmilk.

Cluster 2 contained CD8^+^ T cells with differential expression of the effector chemokine *CCL5* and cytotoxicity-related molecules (Figure 3a), including granzymes (*GZMK/A/B/H*), granulysin (*GNLY*), perforin (*PRF1*), and NK cell granule protein 7 (*NKG7*), as well as the antiviral enzyme *APOBEC3G* (Figure 2C). We confirmed with FACS that breastmilk CD8^+^ memory T cells across all time points produced granzyme B and K, similar to PBMC (Figure 3C and Supplemental Figure 4A). The effector chemokine CCL5 was characteristic of breastmilk, with over 5 times more CCL5-producing memory CD8^+^ T cells compared with PBMC (Figure 3B). In addition, a strong effector function was supported by increased coproduction of granzyme B and K, although only significantly different from PBMC at 6 months postpartum (Supplemental Figure 4B). This effector profile might partially be controlled by *EOMES*, an effector memory transcription factor that was differentially expressed in the CD8^+^ cluster 2 (Figure 3A). With FACS, we showed significantly higher protein-level expression of EOMES in breastmilk CD8^+^ memory T cells compared with PBMC, increasing with time postpartum (Figure 3D).

The CD4^+^ T cell cluster 3 expressed genes related to cytokine/TCR signaling, including *SOS1*, *PAG1*, SOCS2/3, and *CYLD* (Figure 2C). An activated immune cell profile was even more prominent in the predominantly DN cluster 5, with top DEG including *NFKBIA/D/Z*, *NR4A1/2*, *GADD45A/B/G*, *DUSP1/2*, *NFKB2*, *JUN/B/D*, *FOS*, and *IER2/3/5/5L* (Figure 2C). Many of these genes are involved in immediate early response signaling pathways following immune- or stress-related activation. Cluster 6 shared many of cluster 5 DEG, including *DUSP* and *NR4A2*, but not *NFKBIA* (Figure 2C).

**Figure 4:**
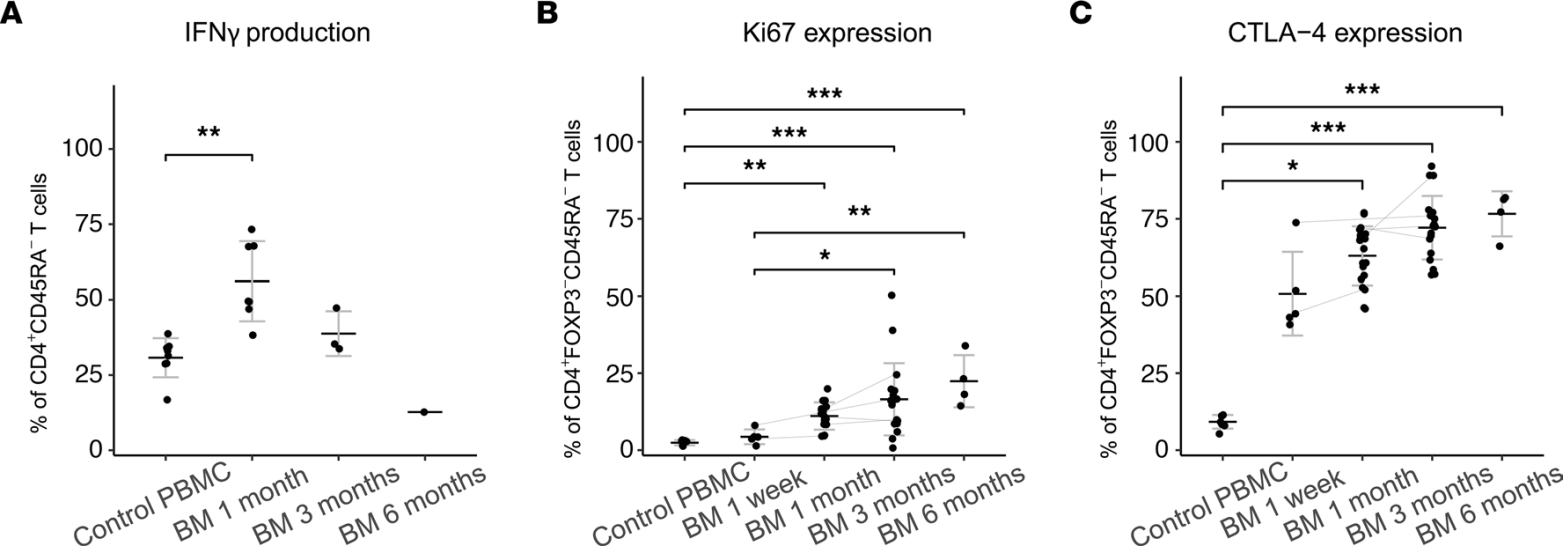
Cytokine production and activated profile of CD4+ breastmilk T cells. (**A**) Frequency of T cells producing IFN-γ upon PMA-ionomycin stimulation, expressed as the percentage of CD4^+^CD45RA^–^ live T cells (PBMC *n* = 8, BM 1 month *n* = 7, BM 3 months *n* = 3, BM 6 months *n* = 1). (**B** and **C**) Frequency of Ki67^+^ (**B**) and CTLA-4^+^ cells (**C**) as the percentage of CD4^+^FOXP3^–^CD45RA^–^ T cells (PBMC *n* = 7, BM 1 week *n* = 5, BM 1 month *n* = 19, BM 3 months *n* = 19, BM 6 months *n* = 4). *P < 0.05, **P < 0.01, ***P < 0.001 with pairwise comparisons among PBMC and BM using the Kruskal-Wallis test followed by Dunn’s test for multiple comparisons. Data represent mean ± SD. Gray lines connect data points of different time points from the same breastmilk donor. BM, breastmilk.

In our FACS data, both CD4^+^ and CD8^+^ breastmilk memory T cells demonstrated cytokine production upon restimulation (Supplemental Figure 4, C and E–G), with significantly increased IFN-γ production at 1 month postpartum for CD4^+^ breastmilk T cells compared with PBMC (Figure 4A). CD4^+^ memory T cells displayed a preferential type-1 immune profile with IFN-γ and TNF-α production, as well as IFN-γ–TNF-α coproduction, but with little IL-13 production (Figure 4A; Supplemental Figure 4, E–G). CD4^+^ memory T cells also demonstrated significantly increased proliferative capacity compared with PBMC, especially at later time points (Figure 4B). In addition, we observed a significantly increased frequency of CTLA-4^+^ memory CD4^+^ and CD8^+^ T cells in breastmilk, compared with PBMC (Figure 4C and Supplemental Figure 4D). The proliferation marker Ki-67 was enriched in CTLA-4^+^ cells (Supplemental Figure 4, H and I), suggesting that activation and regulation go hand in hand in breastmilk T cells.

### Breastmilk is highly enriched for Tregs.

**Figure 5:**
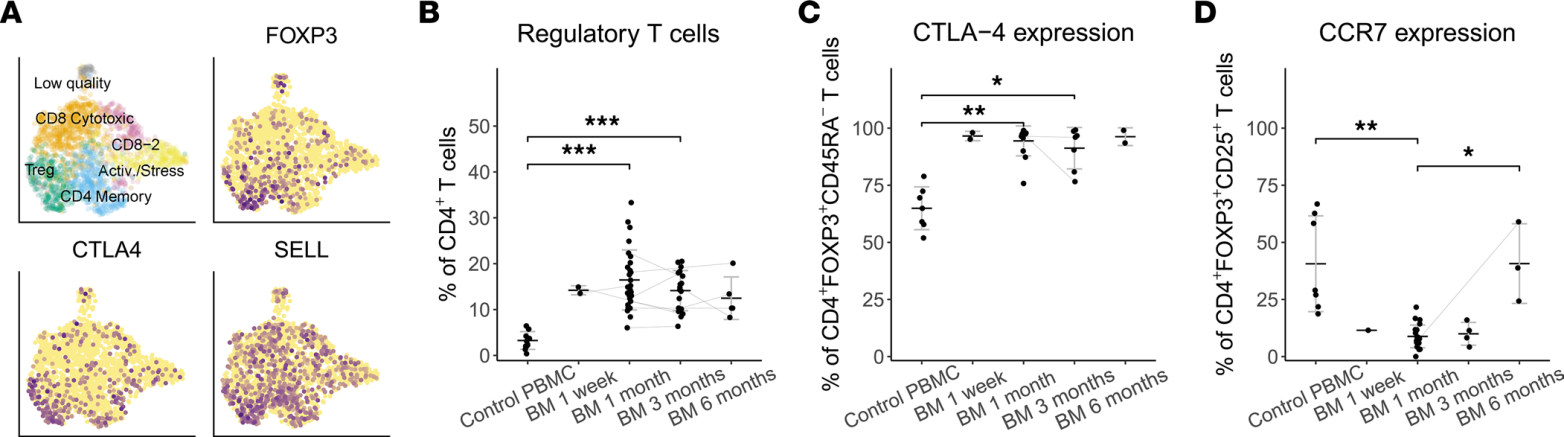
Treg profile in breastmilk. (**A**) UMAP showing gene expression (log-normalized) projections for the cluster 4 characteristic genes FOXP3, CTLA4, and SELL in 1 month postpartum breastmilk (single-cell RNA-Seq). (**B**) Frequency of FOXP3^+^CD25^+^ Tregs expressed as the percentage of CD4^+^ T cells (FACS) (PBMC *n* = 11, BM 1 week *n* = 2, BM 1 month *n* = 28, BM 3 months *n* = 18, BM 6 months *n* = 5). (**C** and **D**) Frequency of CTLA-4^+^ cells as the percentage of CD4^+^FOXP3^+^CD45RA^–^ (PBMC *n* = 7, BM 1 week *n* = 2, BM 1 month *n* = 13, BM 3 months *n* = 7, BM 6 months *n* = 2) and CCR7^+^ cells as the percentage of FOXP3^+^CD25^+^ Tregs (PBMC *n* = 7, BM 1 week *n* = 1, BM 1 month *n* = 23, BM 3 months *n* = 4, BM 6 months *n* = 3) (FACS). FACS data include cells from breastmilk of 1 week and 1, 3, and 6 months postpartum compared with PBMC of age-matched female control donors. *P < 0.05, **P < 0.01, ***P < 0.001 with pairwise comparisons among PBMC and BM across different time points using the Kruskal-Wallis test followed by Dunn’s test for multiple comparisons. Data represent mean ± SD. Gray lines connect data points of different time points from the same breastmilk donor. BM, breastmilk.

Part of the CD4^+^ T cells in our scRNA-Seq data were Tregs (cluster 4), with differential expression of the core Treg markers *FOXP3*, *CD4*, *IL2RA* (CD25), and *CTLA4* (Figure 2C and Figure 5A). Other Treg-related DEG included *TNFRSF1B* (TNFR2), *CD52*, *IL32*, *FCMR,* and *IL10RA*. FACS confirmed that breastmilk is highly enriched for FOXP3^+^CD25^+^ Tregs, with a 3-fold increased frequency compared with PBMC across all time points (Figure 5B). Moreover, expression of the regulatory marker CTLA-4 was significantly increased in breastmilk Tregs (Figure 5C). Whereas differential expression of *SELL* (CD62L) in cluster 4 might indicate lymphoid homing/naivety, we observed low surface expression of CCR7 and CD45RA on breastmilk Tregs (Figure 5D and Supplemental Figure 2K). *SELL* might here instead indicate a more suppressive Treg population (36).

### Breastmilk T cells show signs of recent tissue residence.

**Figure 6:**
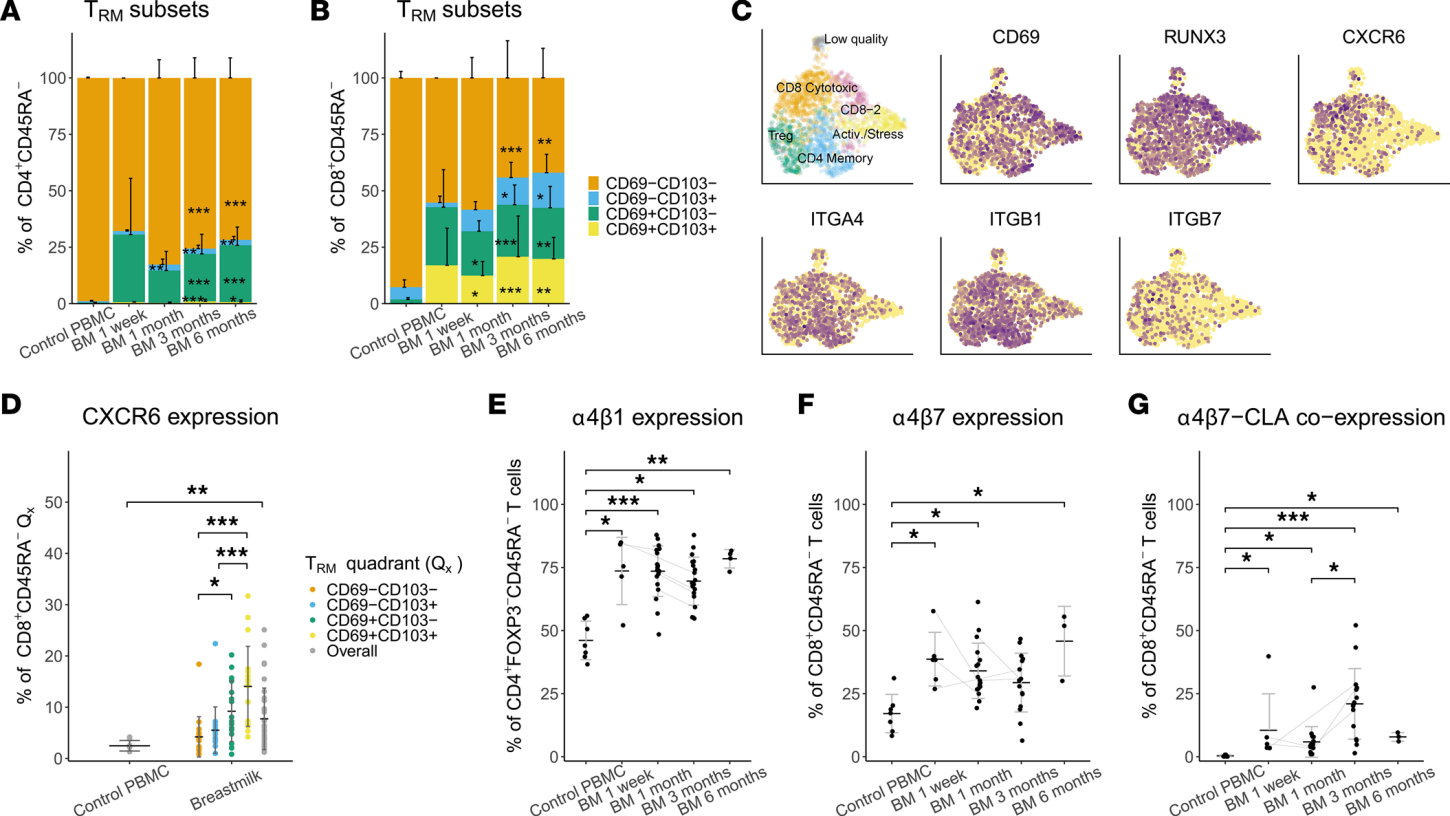
Tissue-residency and tissue-homing profiles in breastmilk T cells. (**A** and **B**) Distribution of tissue-resident memory T cell subsets based on protein expression of the TRM markers CD69 and CD103 (FACS) expressed as the percentage of CD4^+^CD45RA^–^ (PBMC *n* = 8, BM 1 week *n* = 2, BM 1 month *n* = 11, BM 3 months *n* = 17, BM 6 months *n* = 12) (**A**) and CD8^+^CD45RA^–^ T cells (PBMC *n* = 8, BM 1 week *n* = 2, BM 1 month *n* = 11, BM 3 months *n* = 16, BM 6 months *n* = 6) (**B**) with pairwise comparisons between BM time points and PBMC. (**C**) UMAP showing clustering and gene expression (log-normalized) projections for the TRM-related genes CD69, RUNX3, and CXCR6 and the homing receptors integrin α4 (ITGA4), integrin β1 (ITGB1), and integrin β7 (ITGB7) in 1 month postpartum breastmilk (single-cell RNA-Seq, *n* = 7). (**D**) Frequency of CXCR6^+^ cells (FACS) as the percentage of CD8^+^CD45RA^–^ T cells compared between the 4 different CD8^+^ TRM subsets within breastmilk (colored dots) and with the total population in control PBMC (gray dots) (PBMC *n* = 8, BM *n* = 18). (**E**–**G**) Surface expression of different homing receptors (FACS) with pairwise comparisons among PBMC and BM across different time points. Frequencies of α4β1^+^ cells as the percentage of CD4^+^FOXP3^–^CD45RA^–^ T cells (PBMC *n* = 7, BM 1 week *n* = 5, BM 1 month *n* = 19, BM 3 months *n* = 19, BM 6 months *n* = 4) (**E**), α4β7^+^ cells as the percentage of CD8^+^CD45RA^–^ T cells (**F**), and cells double-positive for α4β7 and CLA as the percentage of CD8^+^CD45RA^–^ T cells (**G**). FACS data include cells from breastmilk of 1 week and 1, 3, and 6 months postpartum compared with PBMC of age-matched female control donors (PBMC *n* = 7, BM 1 week *n* = 6, BM 1 month *n* = 17, BM 3 months *n* = 15, BM 6 months *n* = 3). (**A**, **B**, and **E**–**G**) Pairwise comparisons among PBMC and BM time points were tested using the Kruskal-Wallis test followed by Dunn’s test for multiple comparisons. (**D**) For comparisons between breastmilk T cell subsets, the Friedman test was used followed by Bonferroni-corrected pairwise Wilcoxon ranked-sum post hoc testing. *P < 0.05, **P < 0.01, ***P < 0.001. Data represent mean ± SD. Gray lines connect data points of different time points from the same breastmilk donor. TRM, tissue-resident memory T cell; BM, breastmilk.

Our scRNA-Seq data reveal signs of tissue adaptation in breastmilk T cells, with high expression of the tissue-resident memory (TRM) markers *CD69* and *RUNX3* throughout all clusters, but especially in clusters 5 and 6 (Figure 6C). In addition, the TRM-related marker *CXCR6* was differentially expressed in the CD8^+^ cluster 2 (Figure 6C). FACS confirmed that high surface expression of TRM markers (CD69/CD103) distinguished breastmilk from circulating CD4^+^ and CD8^+^ memory T cells, on which these markers are mostly absent (Figures 6, A and B). For CD8^+^, almost half of the CD69^+^ cells coexpressed CD103, with a small population of CD103^+^CD69^–^ cells (Figure 6B). Surface expression of CXCR6 was enriched in the CD69^+^CD103^+^ TRM population and was significantly increased compared with PBMC (Figure 6D). We found that granzyme K production was significantly lower in the CD69^+^CD103^+^ TRM compared with the CD69^–^CD103^–^ population (Supplemental Figure 6C), whereas there was a trend toward higher CCL5 in the TRM population (Supplemental Figure 6D). CD69, but not CD103, expression was also increased in the breastmilk DN and Treg populations, comparable with CD4^+^ memory T cells (Supplemental Figure 6, A and B). The DEG of the CD4^+^ cluster 3 included a set of genes (*CCR6*, *MAF*, *RGS1/2*, and *CREM*) (Supplemental Figure 5A), which have been associated before with skin and intestinal CD4^+^ T cells (37). Gene set enrichment testing of a core human TRM signature (38) showed overlap with the afore-mentioned tissue-related genes in CD4-expressing cluster 3 as well as with CD8A/B-expressing clusters 2 and 6 (Supplemental Figure 5B). Together, these results indicate a tissue origin of both CD4^+^ and CD8^+^ breastmilk memory T cells, possibly originating from the breast tissue itself.

**Figure 7:**
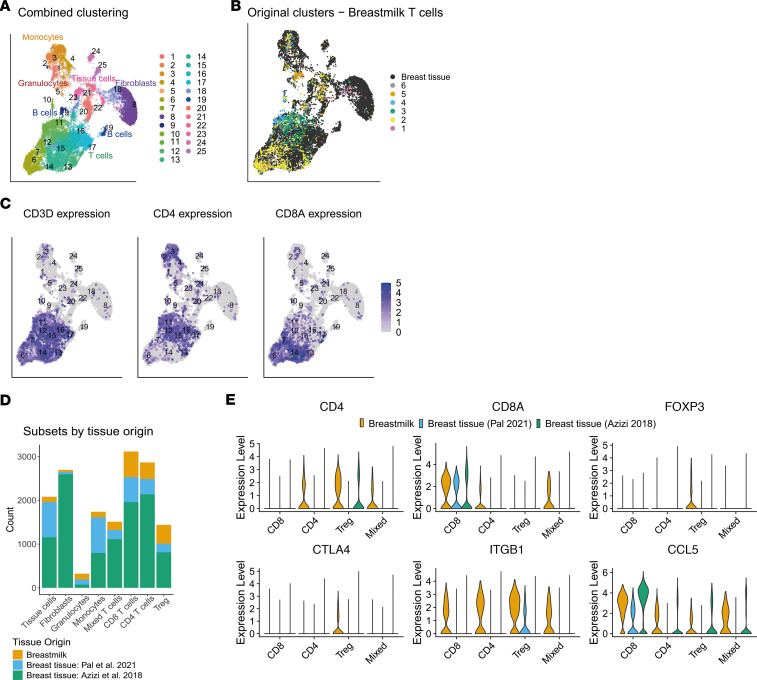
Partial overlap of breastmilk with breast tissue T cells. Clustering of breastmilk T cells with the immune cell subsets from healthy breast tissue extracted from 2 publicly available single-cell RNA-Seq datasets, with breastmilk T cells in orange (*n* = 7), breast tissue cells from Pal et al. (39) in blue (*n* = 12) and breast tissue CD45^+^ immune cells from Azizi et al. (40) in green (*n* = 10). (**A**) UMAP showing cluster annotation. (**B**) UMAP showing the original cluster annotation of the breastmilk T cells (colors) projected onto the combined clustering space of breastmilk and breast tissue (black). (**C**) UMAP gene expression (log-normalized) projections for CD3D, CD4, and CD8A. (**D**) Frequency of each of the 3 datasets for the main cell types identified in **A** and **C**. (**E**) Violin plots comparing gene expression (log-normalized) of CD4, CD8A, FOXP3, CTLA-4, ITGB1 (integrin β1), and CCL5 between the breastmilk and breast tissue datasets for each of the major T cell subsets.

We further explored the potential breast tissue origin of breastmilk T cells by comparing our data with 2 public scRNA-Seq datasets of (immune) cells from healthy breast tissue biopsies (39, 40). After integration, we found that breastmilk T cells clustered mostly together with breast tissue T cells (Figure 7, A, B and D, and Supplemental Figure 7, A–C), with separation into *CD4*-expressing and *CD8*-expressing clusters (Figure 7C). To compare breastmilk and breast tissue T cells, we then performed pseudobulk differential gene expression, yielding 3,907 genes upregulated in milk versus tissue and 1,347 in tissue versus milk (logFC > |1| and adjusted *P* [*P*_adj_] < 0.05; Supplemental Table 3). Even after subsetting for the clusters enriched for T cells, we found that the comparison of breast tissue versus milk was confounded by the presence of many non–T cell–related genes. However, we found some indications that CD8-expressing cells were more similar across milk and tissue than CD4-expressing T cells. Especially many of the Treg-related genes from the above described breastmilk cluster 4 (*FOXP3*, *CTLA4*) appeared to be mostly absent in T cells from breast tissue (Figure 7E). Furthermore, we found significantly higher expression of genes related to homing (*ITGA4*, *ITGB1*, *CCR6*) and cytokine/TCR signaling (e.g., *SOS1*) in T cells from breastmilk compared with breast tissue (Figure 7E and Supplemental Figure 8). Together, these data suggest difference in regulation between breast tissue versus milk residing CD4^+^ T cells.

In contrast, most of the genes related to cytotoxic T cells were similarly expressed between breastmilk and breast tissue T cells, including *CD8A*, *GZMB*, and *CCL5* (Figure 7E and Supplemental Figure 8). The only differences that stood out in CD8-expressing T cells from breastmilk versus tissue were higher expression of *GZMK*, *EOMES*, *CCR5*, and *CXCR6* (Supplemental Figure 8). We found that part of the breastmilk T cells ended up in non–T cell clusters that expressed high levels of stress-related genes such as heat-shock proteins. In general, many of the genes related to activation and/or stress, including *NFKBIA* and *JUN*, were enriched in breast tissue and abundantly expressed across breast tissue T cell and non–T cell clusters, potentially indicating a relation with tissue digestion. In summary, we found partial overlap of breastmilk with breast tissue T cells, indicating a potential tissue-origin of breastmilk T cells, especially for cytotoxic CD8^+^ T cells. However, differences in the expression of genes related to homing and regulation might indicate that these characteristics are more specific for breastmilk T cells.

### Tissue-homing capacities of breastmilk T cells.

Expression of homing receptors would be a prerequisite for a tissue role of breastmilk T cells in either the breast tissue environment or in neonatal immune defense, as indicated by animal studies. Our FACS data show expression of skin-homing (cutaneous leucocyte-associated antigen [CLA]), intestinal-homing (integrin α4β7, CCR9) and general inflammatory homing (integrin α4β1) receptors in breastmilk T cells from all time points (Figure 6, E–G, and Supplemental Figure 6, E–G). Compared with PBMC, breastmilk contained significantly more CLA-, α4β7-, and CCR9-expressing CD8^+^ memory T cells and more CLA- and α4β1-expressing CD4^+^ memory and Tregs (Figure 6, E and F, and Supplemental Figure 6, E–G). Breastmilk also contained significantly more T cells with a broad homing profile, such as α4β7^+^CLA^+^ and α4β1^+^CLA^+^, which are rare in PBMC (Figure 6G). FACS data partially confirm scRNA-Seq data, where *ITGA4* and *ITGB1* were enriched in both CD4^+^ clusters, but *ITGB7* only in the Treg cluster (Figure 6C). Both proliferation (Ki-67) and regulation (CTLA-4) were significantly increased in T cells positive for homing receptors, especially for CLA^+^, but not for CCR9^+^ cells (Supplemental Figures 9 and 10). Together, these data suggest that breastmilk contains T cells with tissue migration potential and that part of these cells appear to be more activated.

### Variation in part of the breastmilk T cell profile correlates with clinical parameters.

**Figure 8:**
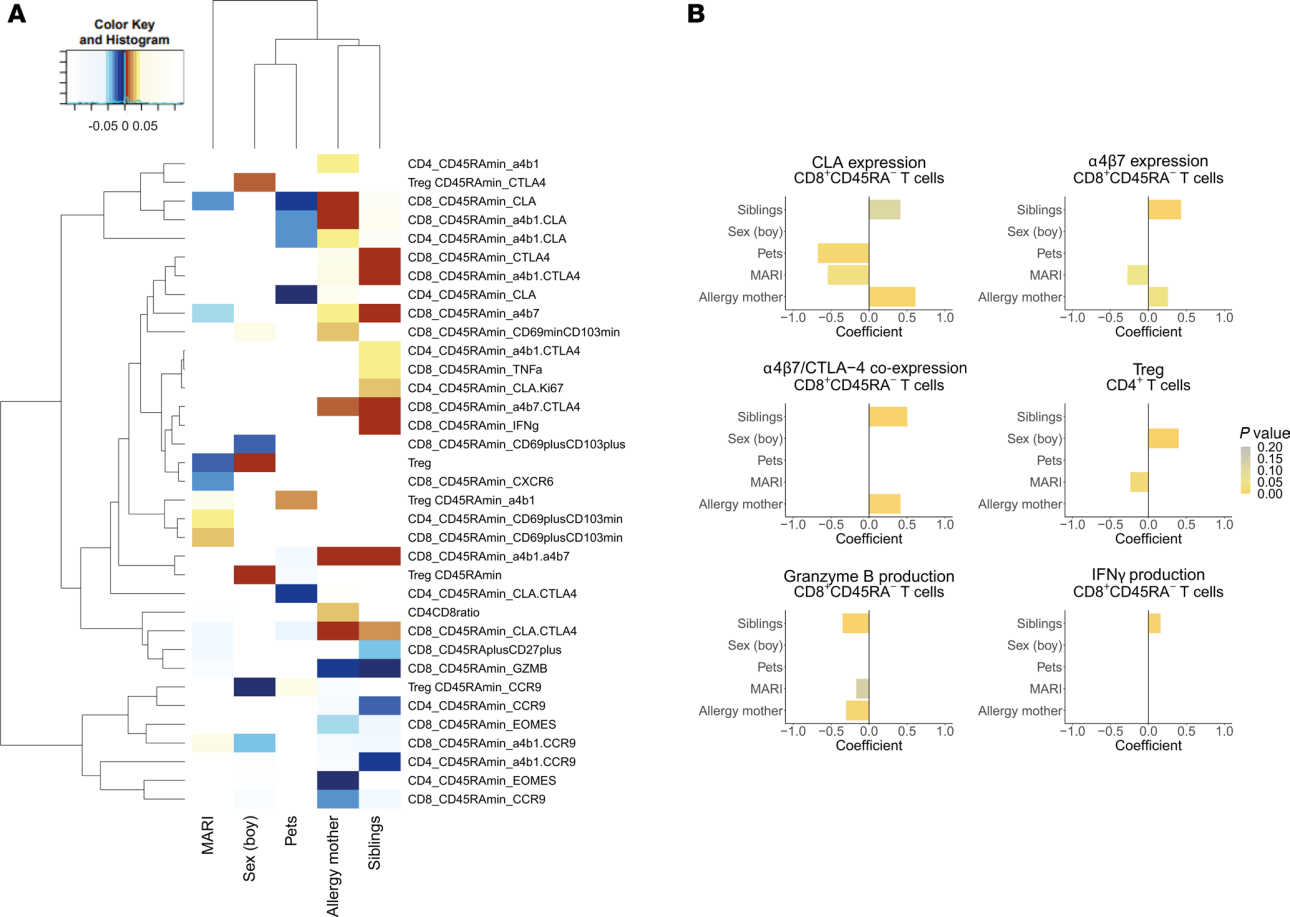
Variation in part of the breastmilk T cell profile correlates to clinical parameters. *n* = 217 BM samples (see Tables 1 and 2 for missing data on clinical parameters). (**A**) Heatmap visualizes a selection of the T cell parameters that were significantly correlated to 1 or multiple clinical parameters, selected by stepwise multiple linear regression on log-transformed T cell parameters. Color intensity indicates the level of significance for the contribution of each clinical parameter on each T cell parameter, with yellow to red indicating a positive relationship, light blue to dark blue a negative relationship, and white indicating that the clinical parameter did not display explanatory power during stepwise model selection. (**B**) Effect size (regression coefficient) for the relation between T cell and clinical parameters, showing a selection of the T cell parameters from **A** that displayed a highly significant correlation to 1 or multiple clinical parameters. Effect size is only shown for those clinical parameters that displayed explanatory power during stepwise model selection. Color intensity indicates the level of significance. All clinical parameters were converted to a binary outcome. MARI, medically attended respiratory infections.

Throughout our data, we observed substantial donor variation in the breastmilk T cell profile. Using stepwise multiple regression, we aimed to discriminate the independent effects of a selection of clinical parameters on the breastmilk T cell profile, with a special interest in respiratory infections of the infant (medically attended acute respiratory infections [MARI]) (Figure 8). We found that allergy of the mother and the presence of 1 or more siblings had most influence on the breastmilk T cell profile (Figure 8A), with a significant positive relationship with expression of most homing receptors (CLA, α4β7, and α4β1), activation markers, and cytokine production but a significant negative relationship to CCR9 expression and a cytotoxic T cell profile (Figure 8B). Contrary to maternal allergy and siblings, the presence of pets had a significant negative correlation with homing receptor expression (Figure 8A). Additionally, we found that nursing a boy was most strongly related to a Treg profile (Figure 8, A and B). When we then considered independently the T cell profile in infants who experienced 1 or more MARI, we found a significantly lower percentage of CD8^+^ T cells expressing the homing receptors CLA and α4β7 (Figure 8B).In addition, MARI were associated with a significantly lower expression of the TRM-related marker CXCR6 but a higher percentage of CD69^+^CD103^–^ T cells. In summary, we found the strongest influence of maternal/household factors on the breastmilk T cell profile but also identified a couple of breastmilk T cell characteristics with a potential relationship to neonatal health.

## Discussion

Infants who are breastfed are less susceptible to severe (respiratory) infections compared with formula-fed infants (4, 5, 41). Here, we performed scRNA-Seq and flow cytometry on breastmilk T cells to get a detailed overview of the different populations and their potential functionality. Our in-depth characterization of breastmilk T cells reveals an activated effector Th1 memory profile and prominent regulation, which was relatively stable throughout lactation. Increased expression of both tissue-residency markers and a broad homing receptor profile suggested that breastmilk T cells execute their effector role in specific tissue sites.

In line with previous FACS studies, our scRNA-Seq data show signs of recent activation in breastmilk T cells, including signaling of the NF-κB and MAPK pathways (23, 25, 28). Although part of this activation-like transcriptional profile might be induced by tissue-digestion stress, we confirmed on a protein-level that breastmilk contains an increased frequency of recently proliferated T cells and T cells capable of producing Type 1–immunity effector molecules. The presence of cytokines and granzymes in breastmilk is well established (42, 43) but has not been demonstrated before, to our knowledge, in breastmilk-derived T cells. We show that production of the chemokine CCL5 is strongly increased in breastmilk, indicating recent TCR-related activation (44, 45).

Throughout our data, we found activation balanced by regulation, with an increased frequency of Tregs and high expression of CTLA-4. Similar results were reported for colostrum versus time-matched postpartum blood (22), suggesting that the breastmilk T cell profile is not just a reflection of the immunosuppressive environment of pregnancy. Comparing our data with public scRNA-Seq datasets of healthy breast tissue, we found Tregs to be characteristic of breastmilk and more rare in breast tissue. As regulation is critical early in life, these data might support a role of breastmilk Tregs in neonatal immune defense and/or development, potentially through delivery of antiinflammatory mediators. Breastmilk has been reported to contain a range of cytokines and soluble receptors with antiinflammatory and tolerance-inducing properties, such as TGF-β, IL-10, soluble IL-1Rα (sIL-1Rα), and sTNF-RI/II (46). Interestingly, higher intracellular TGF-β was found in colostrum compared with blood-derived Tregs (22). However, another study on IL-10 implied that mammary gland epithelial cells were a more likely source than breastmilk leukocytes (47). Future studies should further elucidate to what extent breastmilk Tregs could be involved in the delivery of certain antiinflammatory mediators to the infant or might have a different function.

Guided by enrichment of tissue residency–related gene expression (48) in our scRNA-Seq data, we show surface expression of the TRM markers CD69, CD103, and CXCR6 in breastmilk, which are mostly absent on PBMC. Previous studies also show increased CD103 expression on breastmilk T cells (25, 27, 28) but related this mostly to mucosal homing. Our results now suggest that at least part of the breastmilk T cell population originated from tissue, likely from the breast tissue itself. In our comparison between breastmilk- and breast tissue–derived T cells, we found a large degree of overlap between their gene expression profiles, especially for cytotoxic CD8^+^ T cells. Increased immune cell counts, Th1 cytokine, and CCL5 levels have been reported in unilateral milk of the inflamed breast (17, 49, 50). In addition, the breastmilk TCR repertoire showed little overlap with PBMC (27), indicating a location-specific function. The effector profile we observed might reflect a role in local immune defense against breast tissue infections. In our cohort, we could not substantiate this hypothesis due to the low number of (reported) mastitis cases and inadequate methodology for subclinical detection. However, we did observe a significantly lower percentage of granzyme B^+^ memory CD8^+^ T cells in breastmilk of mothers with multiple children, which might be related to the higher chance of developing mastitis in primiparous women (51).

Beside tissue-residency markers, we showed that breastmilk memory T cells express a range of homing receptors, often in increased frequency compared with PBMC. Moreover, we observed coexpression of different homing receptors, such as CLA and integrin α4β7, which is rare on PBMC. These combinations could indicate a broad homing profile or might reflect a specific homing profile required for entering the breast tissue (52). In our clinical data associations, we observed that homing receptor expression in breastmilk T cells mostly correlated with maternal allergy and the presence of siblings but negatively with the presence of pets. Whether these observations relate to increased breast tissue homing or a different tissue origin/destination of breastmilk T cells remains an outstanding question. In neonatal mice, viable breastmilk CD8^+^ T cells expressing α4β7 and CCR9 were shown to migrate into neonatal Peyer’s patches and intestinal mucosa (29) and produce IFN-γ and granzymes upon stimulation, in contrast to neonatal-origin T cells. However, epithelial barrier development differs strongly between species, with gut closure occurring postpartum in mice but mostly occurring prepartum in humans (53). Thus, it remains unclear to what extent homing receptor expression in breastmilk could be involved in homing to neonatal tissues and immune protection or development in human neonates.

In our cohort, we had the opportunity to follow the breastmilk T cell population over time since birth. Overall, we found remarkably few changes in general composition, cytotoxic profile, regulation, tissue-residency, and homing. We observed some increase in proliferation and EOMES expression toward later breastmilk (3–6 months postpartum), whereas cytokine production was highest in early breastmilk. However, whether these observations relate to functional differences throughout lactation or reflect a technical bias, such as increased fragility of T cells in later milk, remains to be established.

In this study, we lacked the power and study set-up to draw definite conclusions about the functional implications of T cells in breastmilk, but we could show some correlations between the T cell profile and clinical parameters. Larger cohort studies are necessary to further validate these; for instance, validation can be done by investigating the relation between household factors or specific maternal allergies and the distribution of T cells throughout maternal tissues (including breast) and breastmilk, or between the tissue-resident breastmilk T cell profile and local infections of the breast. In addition, human studies using safe labeling approaches such as deuterium-labeling, could be an interesting follow-up to research neonatal absorption of breastmilk T cells and determine whether certain characteristic aspects of the breastmilk T cell profile correlate to neonatal development.

In conclusion, we provided a comprehensive overview of the gene and phenotypic protein expression profile of T cells present in breastmilk over time and observe a tissue-related memory population with broad functional characteristics. With these data, we provide directions for future researchers to further determine the functional relevance of T cells in human breastmilk for infant and/or maternal health.

## Methods

### Sex as a biological variable.

All participants in this study were of the female sex. By including only female participants for control PBMC samples, we strived to have the most comparable setting to breastmilk.

### Study population and sample collection.

Samples and clinical data were collected and processed as described by van Stigt et al. (34). In total, 217 samples were collected from 139 mothers, including 1 mother with twins. Baseline characteristics of the infants, relating to birth, household composition, and clinical follow-up during the first year of life, are summarized in Table 1. Since self-reported ethnicity had a high level of missing data, this variable was left out of the analysis. Parental characteristics, relating to the pregnancy and parental allergic disease, are summarized in Table 2. The majority of infants were born full-term following healthy pregnancy (39.8 ± 1.5 weeks; 26% cesarean section) with normal birth weight (3,534.4 ± 554.5 g) and reassuring Apgar score (scores 7–10). Primary outcome in the overarching PRIMA study was the number of MARI, defined as parent-reported medical visits for infant respiratory illness during first year of life (45% infants were reported with 1 or more MARI).

Breastmilk was collected at 1 week (*n* = 11), 1 month (*n* = 97), 3 months (*n* = 74), and/or 6 months (*n* = 35) postpartum, with 23 mothers who donated at 2 or more time points. Depending on the immune cell isolation yield, 1 or more experiments were performed with each breastmilk sample. An overview of the samples used per experiment can be found in Supplemental Table 1 as well as in the Supporting Data Values file.

Control peripheral blood (*n* = 8 females, 34.1 ± 7.6 years) was obtained through the employer donor service of the University Medical Centre Utrecht.

### Immune cell isolation.

Breastmilk samples were stored (maximum 24 hours) and transferred cold until isolation. Milk was centrifuged twice (10 minutes, 600*g*) to first separate aqueous from cream layer and then, after dilution in PBS (Sigma-Aldrich), cream layer from immune cell pellet. The cell pellet was analyzed by FACS on the same day or cryopreserved for later detection of cytokine production capacities.

Whole blood was collected in sodium-heparin–coated tubes. PBMC were isolated using Ficoll-Paque density gradient centrifugation (580*g*, GE Healthcare) and cryopreserved in liquid nitrogen (RPMI medium, Thermo Fisher Scientific; 20% FCS, Invitrogen; 1% L-glutamin, Thermo Fisher Scientific; 1% penicillin/streptomycin, Sigma-Aldrich; 10% DMSO, Sigma-Aldrich).

### Flow cytometry.

Cells were stained with surface antibodies (Supplemental Table 2) in PBS with 2% FCS (Invitrogen), 0.1% natrium azide (Sigma-Aldrich), 2% FcR blocking reagent (Miltenyi Biotec), and 8% Brilliant Staining Buffer (BD Biosciences). For part of the FACS panels, cells were stained with a fixable viability dye (eFluor 506; eBioscience) in PBS. For intracellular staining, cells were fixated (Cell Fixation and Permeabilization Kit, Invitrogen) and then stained in dH_2_O with Permeabilization Buffer (eBioscience) and 2% FcR blocking reagent (Miltenyi Biotec). Samples were measured with the LSR Fortessa (BD Biosciences) flow cytometer in combination with FACS DIVA software (BD Biosciences).

For intracellular cytokine detection, cells were stimulated with 20 ng/mL PMA (Sigma-Aldrich) and 1 μg/mL ionomycin (Calbiochem) in RPMI medium with 10% FCS (Invitrogen), 1% L-glutamin, and 1% penicillin/streptomycin (240 minutes, 37°C), with addition of 0.07% protein transport inhibitor (BD Golgi stop) at 30 minutes.

### Statistics.

Flow cytometry data were analyzed using FlowJo software (version 10.18), including only samples with > 75 cells in the subset of interest for comparisons with PBMC or > 50 cells for comparisons between breastmilk T cell subsets. Statistics and graphics (54) were performed in R version 4.2 (55). Marker expression was compared within memory (CD45RA^–^) T cells, unless mentioned otherwise. Breastmilk from different time points and PBMC were compared for a significant between-group difference (*P* < 0.05) using the Kruskal-Wallis test followed by Dunn’s test for multiple comparisons (56, 57). Because of the low number of time point follow-up data, we did not correct for paired samples from the same donor. For comparisons between breastmilk T cell subsets, the Friedman test was used followed by Bonferroni-corrected pairwise Wilcoxon rank sum post-hoc testing. To test to which extent clinical parameters could explain variation in breastmilk T cell marker expression, we performed stepwise multiple linear regression for each T cell marker (log-transformed) to select clinical variables with explanatory power.

For scRNA-Seq, 1 month postpartum breastmilk samples of 7 donors were used, without overlap with the donors used for the FACS experiments. Live CD3^+^ cells (376 cells/donor) were sorted with the FACS Aria III into 384-wells plates (Bio-Rad) with 5 μL of vapor-lock (QIAGEN) containing 100–200 nL of reverse transcription primers, dNTPs, and synthetic mRNA Spike-Ins (Single Cell Discoveries). Plates were immediately spun down after sorting and frozen to −80°C. Cells were prepared for SORT-Seq as previously described (58). Illumina sequencing libraries were prepared with the TruSeq small RNA primers (Illumina) and were sequenced paired-end at 75 bp read length with 75,000 reads per cell on a NextSeq500 platform (Illumina). Sequencing reads were mapped against the reference human genome (GRCh38) with Burrow-Wheeler Aligner (BWA).

Cells were stained for surface antibodies (Supplemental Table 2) using the same protocol as described for FACS and using the SYTOX blue dead cell stain (Invitrogen) for viability.

The unique molecular identifiers–corrected (UMI-corrected) read counts of the individual samples were analyzed in Seurat v4 (59), performing quality control following the distributor’s outline. SCTransform was used for normalization and variance stabilization, correcting for cell cycle, mitochondrial, and ribosomal gene percentage. Individual samples were integrated and corrected for batch effects using the canonical correlation analysis (CCA) algorithm to find integration anchors. K-nearest neighbor batch effect test (kBET) determined a reduction in batch-effect after integration (60).

K-nearest neighbors were calculated based on the first 30 PCAs, and clustering was performed on the shared nearest neighbors (SNN) graph at a resolution of 0.8, resulting in 6 clusters. Differential gene expression testing between clusters was performed using the MAST test (*P*_adj_ < 0.05). For visualizations, the log-normalized corrected RNA counts in the data assay were used. Gene set enrichment analysis was performed with gene sets derived from literature mentioned in the text, using the AUCell package (61).

For surface protein expression of sorted cells, index-sort FACS data were modified to a value of 0–5, where 0 represents “marker-negative” cells, based on their MFI respective to FlowJo gating, and 1–5 as “marker-positive” cells, divided on quantile MFI expression.

We compared our scRNA-Seq data of breastmilk T cells with 2 publicly available datasets of total (39) or CD45^+^ sorted (40) single cells from healthy breast tissue biopsies. For both tissue datasets, QC and SCT normalization was performed on individual samples in Seurat v4, after which samples were integrated into a single Seurat object per dataset using the CCA-based algorithm. For the dataset from Pal et al. (39), clustering and differential gene expression were then performed as described above in order to select putative immune cell clusters. For all 3 integrated datasets (from milk and tissue), TCR genes were removed and SCT was rerun, after which the 3 datasets containing immune cells from breastmilk or tissue were integrated using the CCA-based algorithm. Clustering was performed at resolution 1.0, resulting in 25 clusters. Based on the results of differential gene expression testing (MAST), the putative T cell clusters were selected and counts were aggregated per sample. Aggregated counts were then used for pseudobulk differential gene expression analysis using DESeq2 (62) to compare breast milk with breast tissue.

### Study approval.

The PRIMA cohort has been approved by the Medical Research Ethics Committee of the University Medical Centre Utrecht, Netherlands (SL/avd/20/500151, protocol 20-578/X-D). All participating parents gave written consent to participate in this cohort.

### Data availability.

All data for reproducing the figures based on FACS and clinical data are available in the Supporting Data Values file. The scRNA-Seq data of 1 month postpartum breastmilk T cells presented in this publication have been deposited in NCBI’s Gene Expression Omnibus (GEO) (63) and are accessible through GEO series accession no. GSE273105. Breast tissue scRNA-Seq data from Pal et al. (39) are accessible through GEO series accession no. GSE161529, and data from Azizi et al. (40) are accessible through GEO series accession no. GSE114727.

## Author contributions

ESS analyzed data, wrote the manuscript with input from all authors, conducted experiments, and contributed to research design; AHVS was leading in setting up the research cohort, was responsible for sample collection and processing, and analyzed data; BL initiated research design, conducted experiments, and analyzed data; ESS, AHVS, and BL were assigned coauthorship in this order based on contribution to writing the manuscript; LL analyzed data, conducted experiments, and contributed to research design; AHH performed sample collection and processing; MMVDW conducted experiments; LJB was responsible for research planning and design and for supervision; JHWL was responsible for research planning and supervision; BVL initiated the research idea and was responsible for research planning and design and for supervision; FVW initiated the research idea, was responsible for planning and design, conducted manuscript set-up together with ESS, and was responsible for supervision. PRIMA group was involved in study design.

## Supplementary Material

Supplemental data

Supplemental table 1

Supplemental table 3

Supporting data values

## Figures and Tables

**Table 1:**
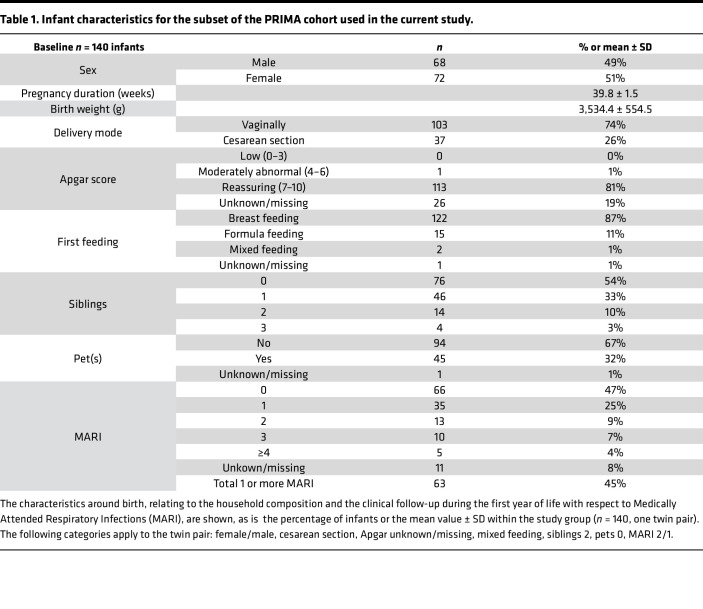
Infant characteristics for the subset of the PRIMA cohort used in the current study.

**Table 2:**
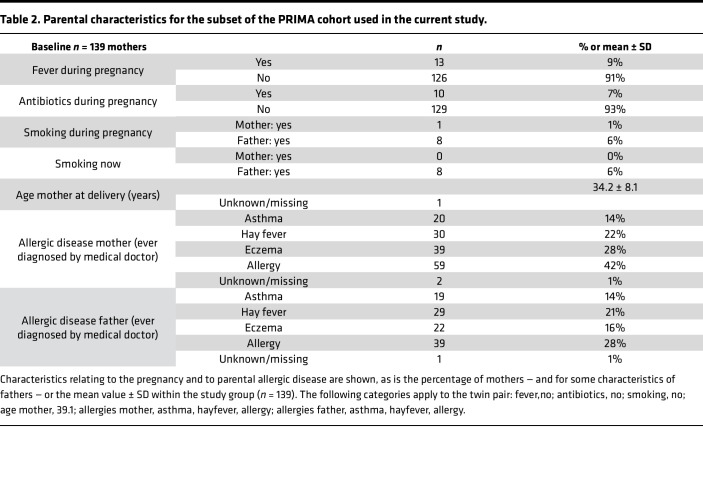
Parental characteristics for the subset of the PRIMA cohort used in the current study.

## Version 1 10/22/2024

Electronic publication
