# Supplementary material for: Human breastmilk memory T cells throughout lactation manifest activated tissue-oriented profile with prominent regulation
Source: JCI Insight. 2024 Sep 3;9(20):e181788. doi: 10.1172/jci.insight.181788 (PMC11530127; doi:10.1172/jci.insight.181788)
Supplement: Supplemental data [file jciinsight-9-181788-s154.pdf]

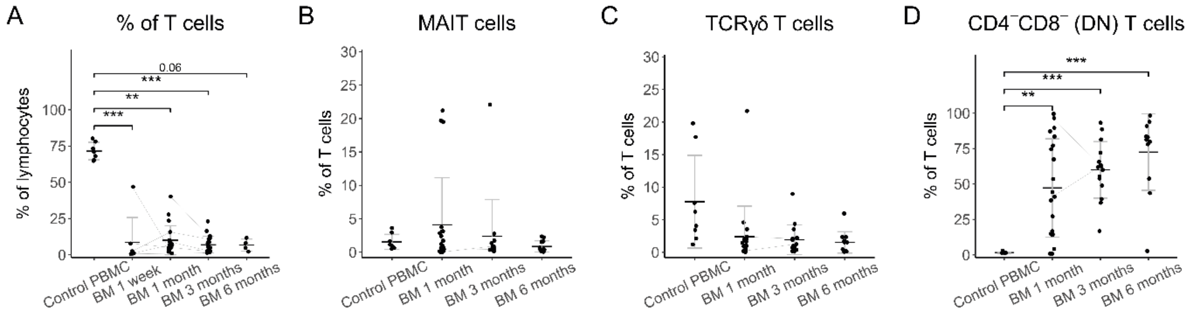

Figure S1: General T-cell composition (FACS) in human breastmilk of 1 week, 1, 3 and 6 months postpartum compared to PBMC of age-matched female control donors. **A)** Frequency of $CD3^{+}$ T cells as a percentage of the lymphocyte gate (PBMC n=7, BM 1 week n=7, BM 1 month n=19, BM 3 months n=20, BM 6 months n=4). **B)** Frequency of Mucosal Associated Invariant T cells ($TCRV\alpha7.2^{+}CD161^{+}$) as the percentage of live T cells. **C)** Frequency of T cells with a γδ-TCR as the percentage of live T cells. **D)** Frequency of $CD4^{-}CD8^{-}$ (DN) T cells as the percentage of T cells, excluding MAIT cells and $TCR\gamma\delta^{+}$ T cells. B-D) PBMC n=8, BM 1 month n=20, BM 3 months n=15, BM 6 months n=12. Significance stars denotate if **=$p<0.01$, ***=$p<0.001$ with pairwise comparisons among PBMC and BM across different time points using the Kruskal-Wallis test followed by Dunn’s test for multiple comparisons. Data represent mean±SD. Transparent lines connect datapoints of different time points postpartum from the same breastmilk donor. MAIT: musocal-associated invariant T cells ($TCRV\alpha7.2^{+}CD161^{+}$), BM: breastmilk, DN: double negative, TCR: T cell receptor.

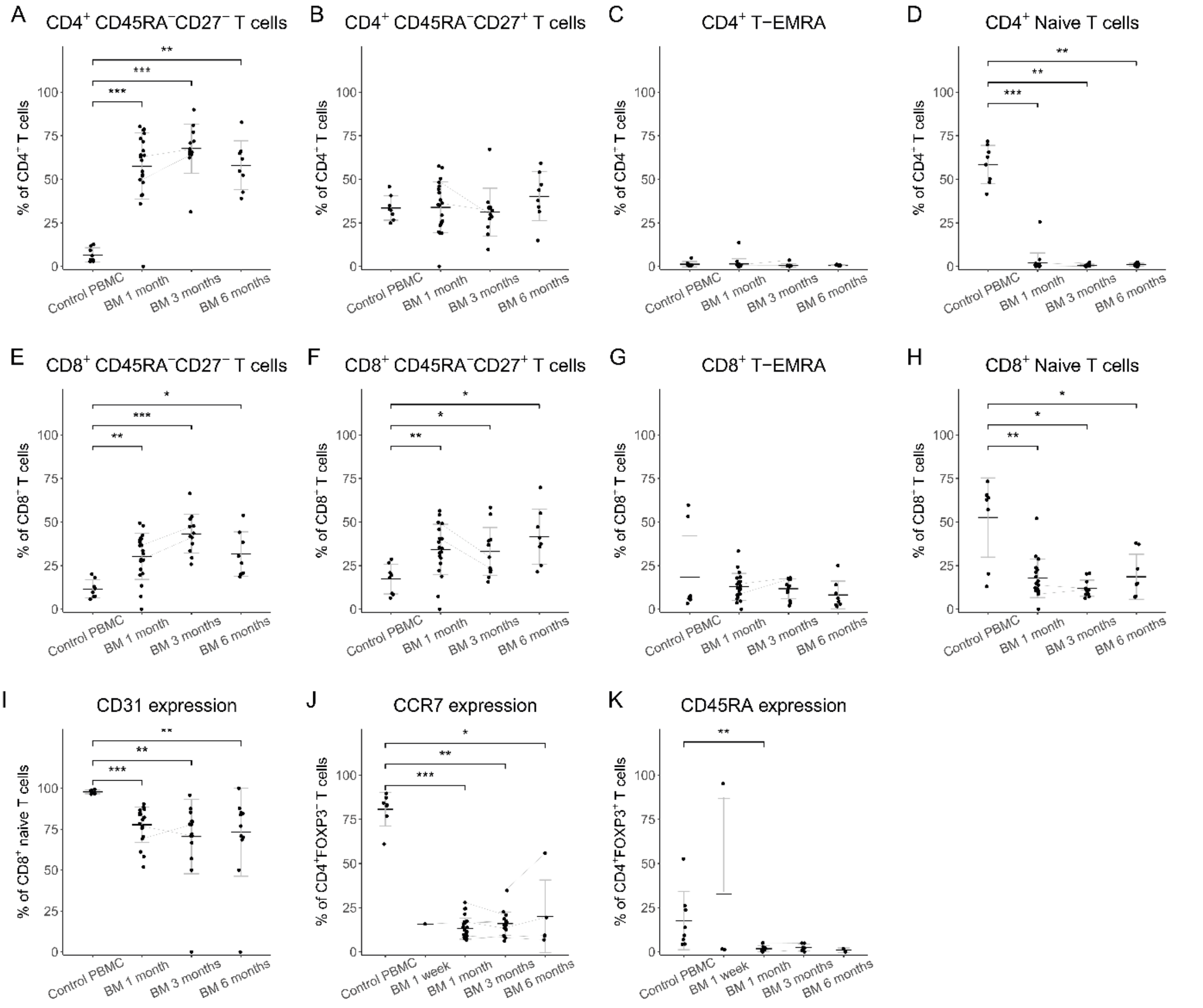

Figure S2: Naïve/Memory differentiation (FACS) of human breastmilk T cells of 1 week, 1, 3 and 6 months postpartum milk compared to PBMC of age-matched female control donors. Frequency of **A)** CD45RA$^-$CD27$^-$, **B)** CD45RA$^-$CD27$^+$, **C)** CD45RA$^+$CD27$^-$ 'T-EMRA' and **D)** CD45RA$^+$CD27$^+$ 'Naive' T cells as the percentage of CD4$^+$ T cells. Frequency of **E)** CD45RA$^-$CD27$^-$, **F)** CD45RA$^-$CD27$^+$, **G)** CD45RA$^+$CD27$^-$ 'T-EMRA' and **H)** CD45RA$^+$CD27$^+$ 'Naive' T cells as the percentage of CD8$^+$ T cells. **I)** Frequency of CD31$^+$ T cells as the percentage of CD8$^+$CD45RA$^+$CD27$^+$ 'Naïve' T cells. A-I) PBMC n=7, BM 1 week n=8, BM 1 month n=20, BM 3 months n=15, BM 6 months n=12. **J)** Frequency of CCR7$^+$ T cells as the percentage of FOXP3$^-$CD4$^+$ T cells (PBMC n=7, BM 1 week n=1, BM 1 month n=27, BM 3 months n=17, BM 6 months n=5). **K)** Frequency of CD45RA$^+$ T cells as the percentage of FOXP3$^+$CD4$^+$ 'Treg' T cells (PBMC n=7, BM 1 week n=2, BM 1 month n=13, BM 3 months n=7, BM 6 months n=2). Significance stars denotate if *=$p<0.05$, **=$p<0.01$, ***=$p<0.001$ with pairwise comparisons among PBMC and BM across different time points using the Kruskal-Wallis test followed by Dunn's test for multiple comparisons. Data represent mean±SD. Transparent lines connect datapoints of different time points postpartum from the same breastmilk donor. BM: breastmilk.

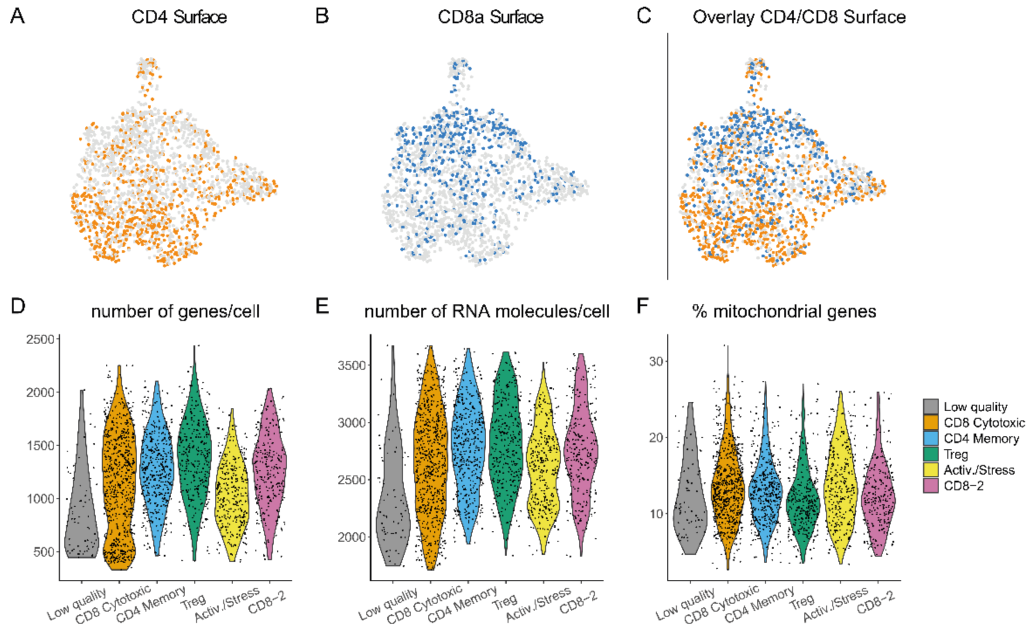

Figure S3: Descriptive figures of scRNAseq data of T cells from 1 month postpartum breastmilk (n=7). UMAP showing index sort data for **A)** surface CD4 (orange) and **B)** surface CD8α (blue), overlayed in **(C)**. Violinplots of quality control metrics, showing **D)** the number of genes (nFeature), **E)** number of RNA molecules (nCount) and **F)** percentage of mitochondrial genes per cell for each of the scRNAseq clusters.

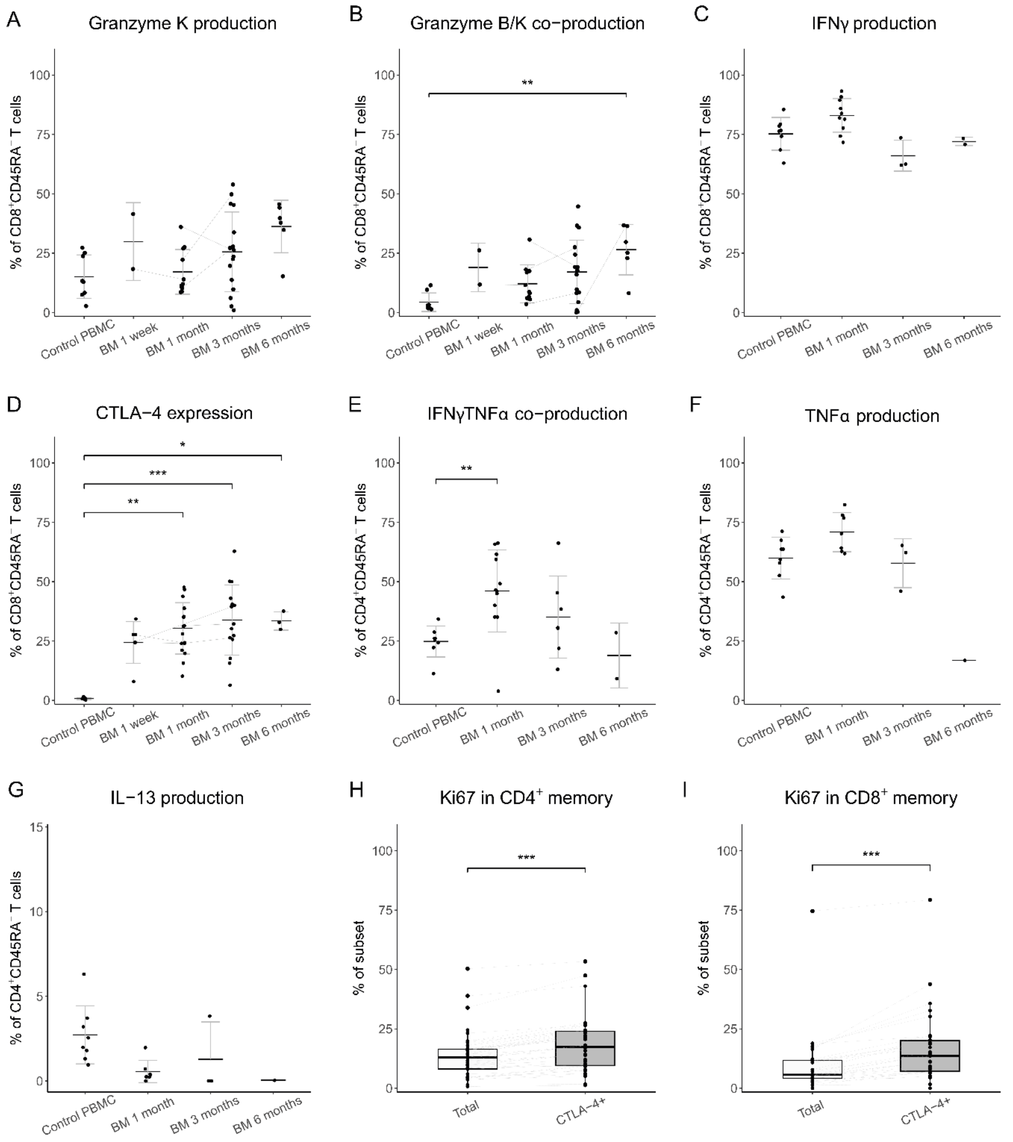

Figure S4: Functional profile of $CD4^+$ and $CD8^+$ memory T cells (FACS) in human breastmilk of 1 week, 1, 3 and 6 months postpartum compared to PBMC of age-matched female control donors. **A-D)** Frequency of granzyme K^+ (PBMC n=8, BM 1 week n=1, BM 1 month n=11, BM 3 months n=16, BM 6 months n=6) **(A)**, granzyme B^+K^+ (PBMC n=8, BM 1 week n=2, BM 1 month n=11, BM 3 months n=17, BM 6 months n=6) **(B)**, $IFN\gamma^+$ (PBMC n=8, BM 1 month n=10, BM 3 months n=3, BM 6 months n=2) **(C)** and CTLA-4^+ (PBMC n=7, BM 1 week n=6, BM 1 month n=17, BM 3 months n=15, BM 6 months n=3) **(D)** cells as the percentage of $CD8^+CD45RA^-$ T cells. **E-G)** Frequency of $IFN\gamma^+TNF\alpha^+$ (PBMC n=8, BM 1 month n=12, BM 3 months n=7, BM 6 months n=2) **(E)**, $TNF\alpha^+$ **(F)** and IL-13^+ **(G)** cells (E-G: PBMC n=8, BM 1 month n=7, BM 3 months n=3, BM 6 months n=2) as the percentage of $CD4^+CD45RA^-$T cells. **H-I)** Frequency of $Ki67^+$ cells within $CD4^+CD45RA^+$ (n=47) **(H)** and $CD8^+CD45RA^+$ (n=41) **(I)** breastmilk T cells, comparing the total population with the subset of cells positive for the immune-checkpoint receptor CTLA-4. Cytokine production was measured following stimulation with PMA/ionomycin. Significance stars denotate if *=p<0.05, **=p<0.01, ***=p<0.001. A-G) Testing pairwise comparisons between T cell subsets within each donor using the Kruskal-Wallis test followed by Dunn's test for multiple comparisons. H-I) Testing paired difference between expression in the total T-cell population and T-cell subset using Wilcoxon rank-sum test. Data represent mean±SD. Transparent lines connect datapoints from the same breastmilk donor. Only samples with more than 50 cells within the CTLA-4^+ populations are included. BM: breastmilk.

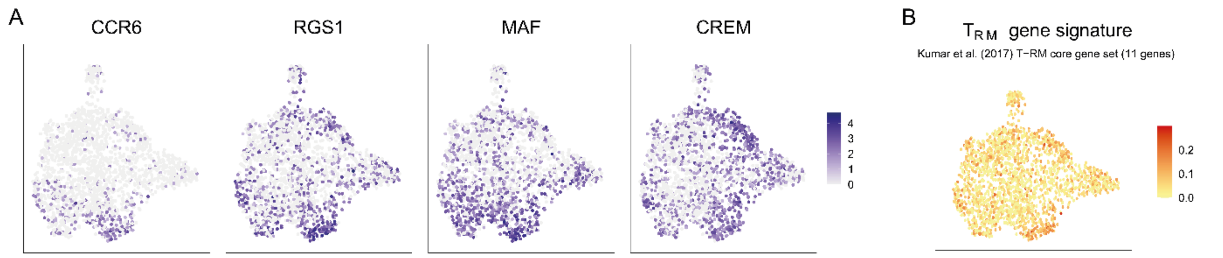

Figure S5: Tissue-related gene expression signature in human breastmilk T cells (n=7). **A)** UMAP showing gene expression (log-normalized) projections of tissue-related genes that ranked high among the DEG in cluster 3 (CCR6, RGS1, MAF, CREM) and cluster 6 (CREM). **B)** UMAP showing gene-set enrichment score (AUCell) of a core human T-RM gene set as published by Kumar et al. (2017). T-RM: tissue-resident memory T cell.

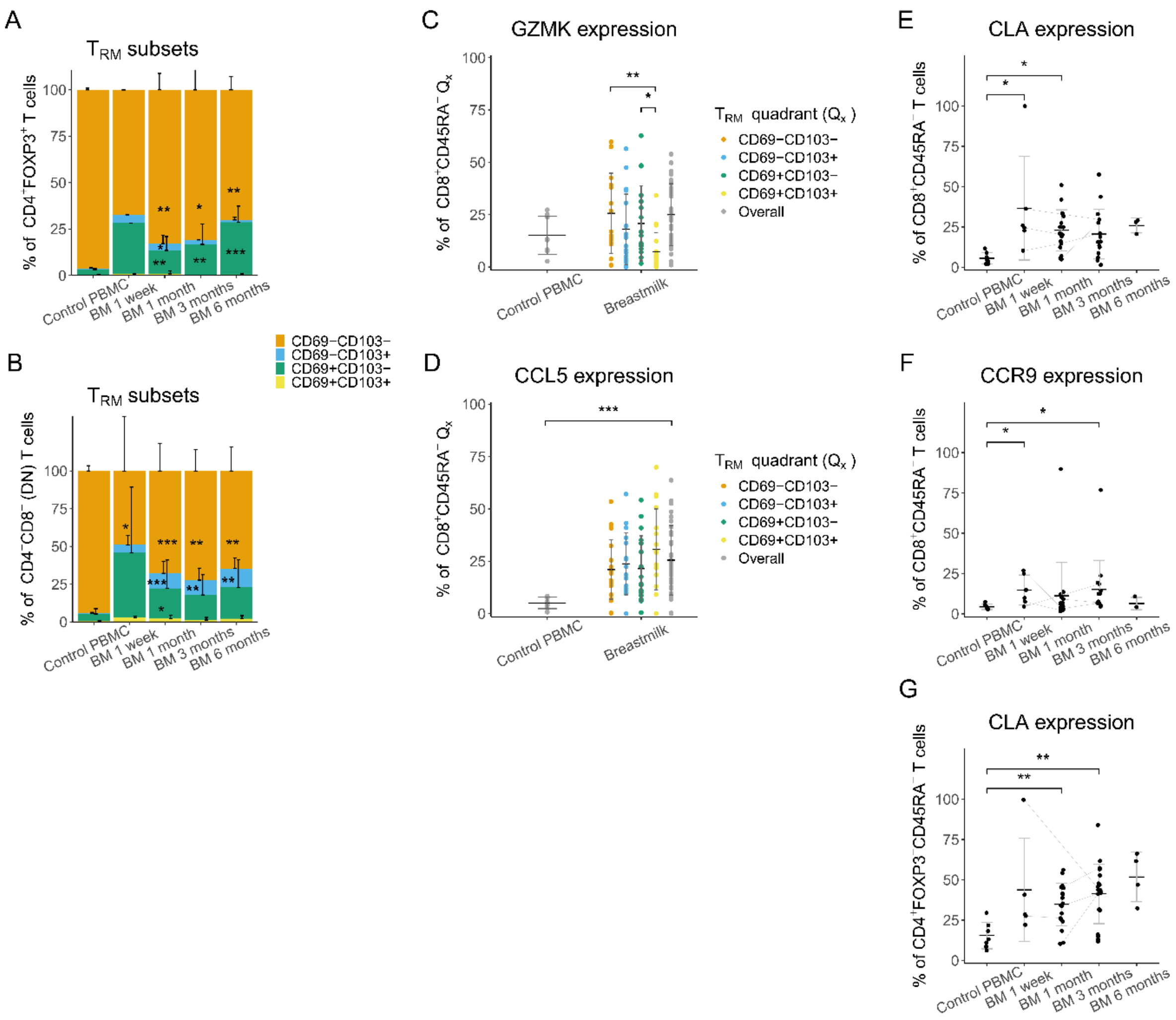

Figure S6: Tissue-residency and tissue homing T cell profiles (FACS) in human breastmilk of 1 week, 1, 3 and 6 months postpartum compared to PBMC of age-matched female control donors. **A-B)** Distribution of different tissue-resident memory T cell (T-RM) subsets based on CD69 and CD103 expression (FACS) as the percentage of $CD4^+FOXP3^+$ T cells (PBMC n=7, BM 1 week n=1, BM 1 month n=23, BM 3 months n=4, BM 6 months n=3) **(A)** and $CD4^-CD8^-$ (DN) T cells (PBMC n=8, BM 1 week n=2, BM 1 month n=28, BM 3 months n=18, BM 6 months n=5) **(B)**. **C)** Frequency of $CCL5^+$ (PBMC n=9, BM n=18) and **D)** granzyme K^+ cells (PBMC n=8, BM n=34) expressed as the percentage of $CD8^+CD45RA^-$ T cells compared between the four different $CD8^+$ T-RM subsets within breastmilk (coloured dots) and with the total population in control PBMC (grey dots). **E)** Frequency of CLA^+ and **F)** $CCR9^+$ cells as the percentage of $CD8^+CD45RA^-$ T cells (PBMC n=7, BM 1 week n=6, BM 1 month n=17, BM 3 months n=15, BM 6 months n=3) and **G)** CLA^+ cells as the percentage of $CD4^+FOXP3^-CD45RA^-$ T cells (PBMC n=7, BM 1 week n=5, BM 1 month n=19, BM 3 months n=19, BM 6 months n=4). Significance stars denotate if *=p<0.05, **=p<0.01, ***=p<0.001. A-B,E-G) Testing pairwise comparisons among PBMC and BM timepoints using the Kruskal-Wallis test followed by Dunn's test for multiple comparisons. D) Testing comparisons between breastmilk T-cell subsets using the Friedman test followed by Bonferroni-corrected pairwise Wilcoxon rank sum post-hoc testing. Data represent mean±SD. Transparent lines connect datapoints of different time points from the same breastmilk donor. BM: breastmilk, T-RM: tissue-resident memory T cell.

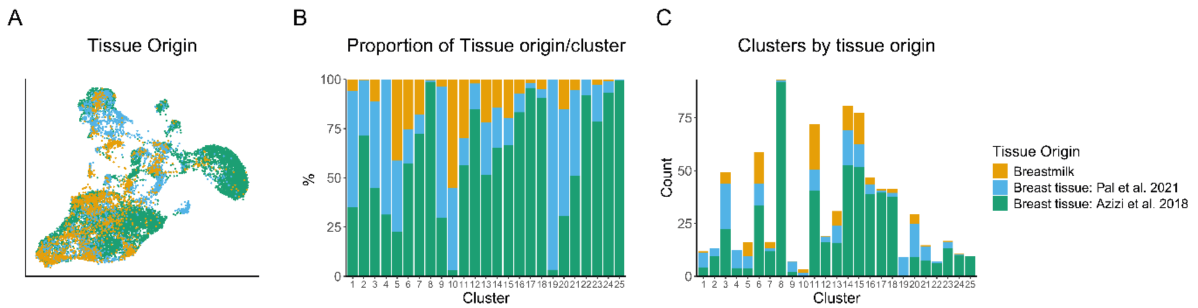

Figure S7: Clustering of breastmilk T cells with the immune cell subsets from healthy breast tissue extracted from two publicly available scRNAseq datasets, displaying breastmilk T cells in orange (n=7), breast tissue cells from Pal et al. (2021) in blue (n=12) and breast tissue CD45^{+} immune cells from Azizi et al (2018) in green (n=10). **A)** UMAP showing localization of the cells originating of each of the three datasets. **B)** Relative and **C)** Absolute frequency of each of the three datasets per cluster.

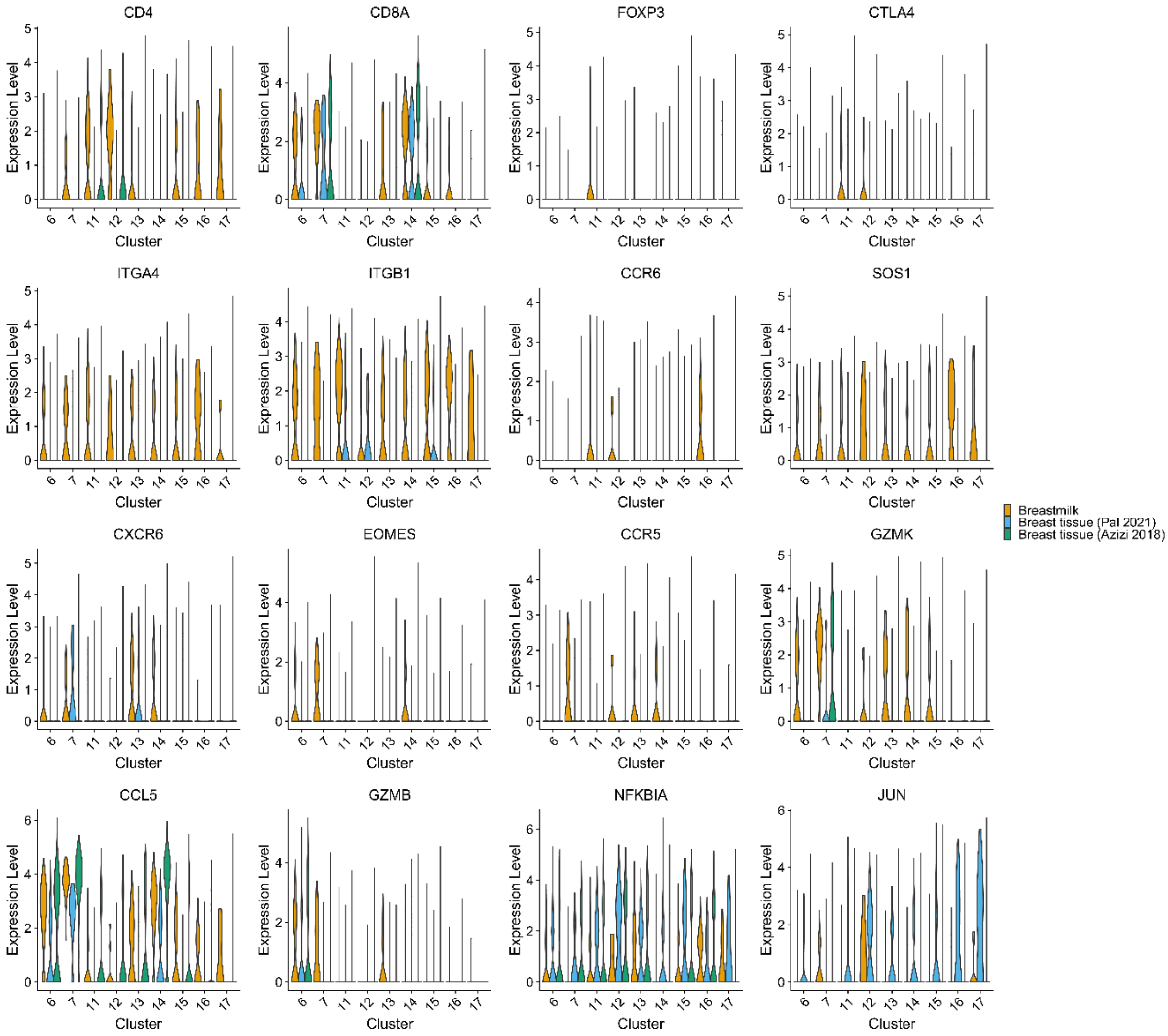

Figure S8: Comparison of gene expression levels of different T-cell markers across breastmilk and breast tissue. Showing violin plots of gene expression (log-normalized) from the clusters enriched for T cells upon clustering of breastmilk T cells together with breast tissue immune cells from two publicly available scRNAseq datasets, with breastmilk T cells in orange (n=7), breast tissue cells from Pal et al. (2021) in blue (n=12) and breast tissue CD45⁺ immune cells from Azizi et al (2018) in green (n=10).

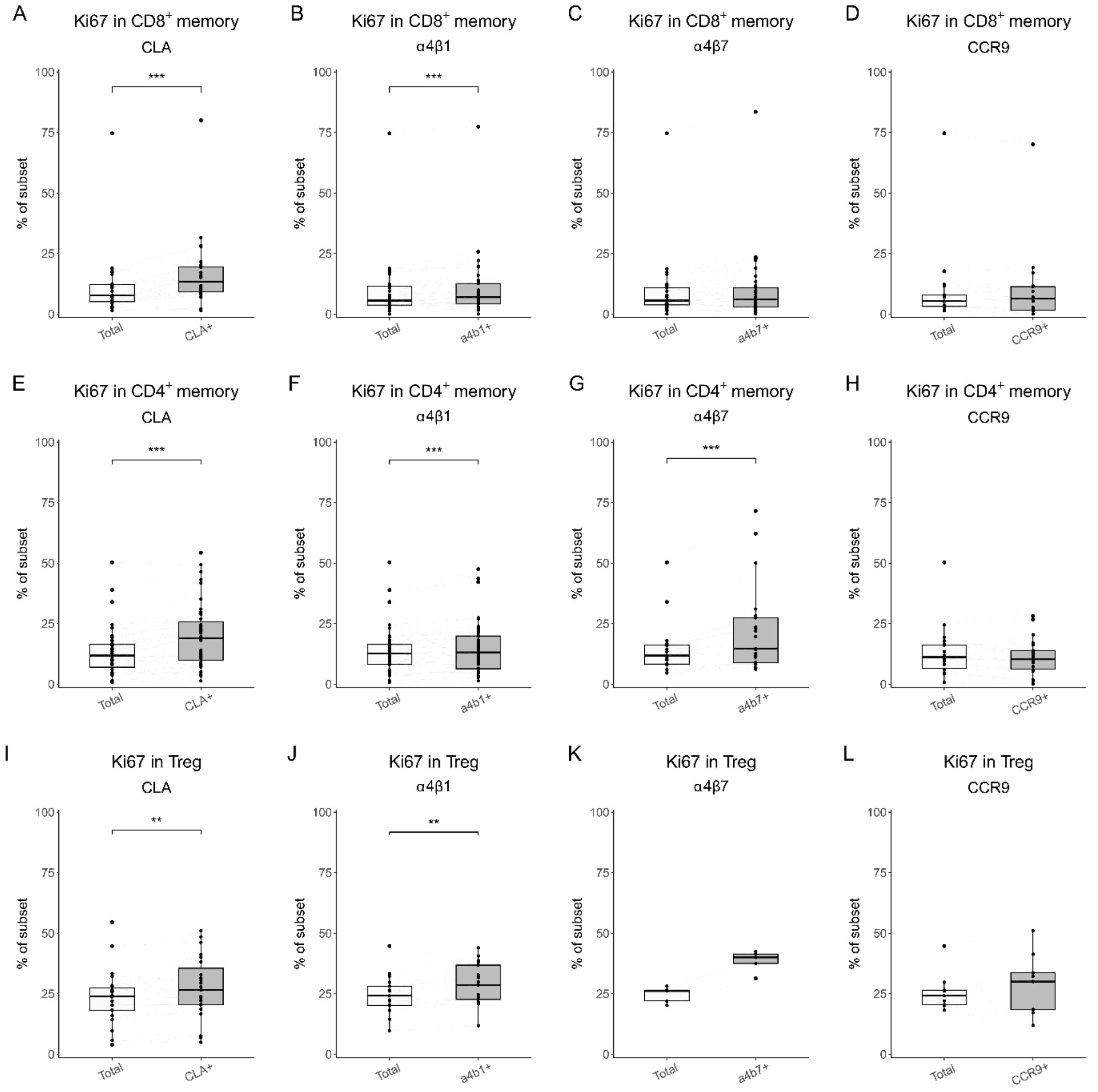

Figure S9: Proliferation of breastmilk T cells, assessed by expression of Ki-67, comparing T cells expressing homing receptors with total T cells (FACS, n=26). Frequency of Ki67$^+$ cells within CD8$^+$CD45RA$^+$ **(A-D)**, CD4$^+$CD45RA$^+$ **(E-H)** and regulatory **(I-L)** breastmilk T cells comparing the total population with the subset of T cells positive for the homing receptors CLA **(A, E, I)**, α4β1 **(B, F, J)**, α4β7 **(C,G,K)** and CCR9 **(D,H,L)**. Significance stars denotate if **=$p<0.01$, ***=$p<0.001$, testing paired difference between expression in the total T-cell population and T-cell subset using Wilcoxon rank-sum test. Transparent lines connect datapoints from the same breastmilk donor. Only samples with more than 50 cells within the homing receptor-positive populations were included.

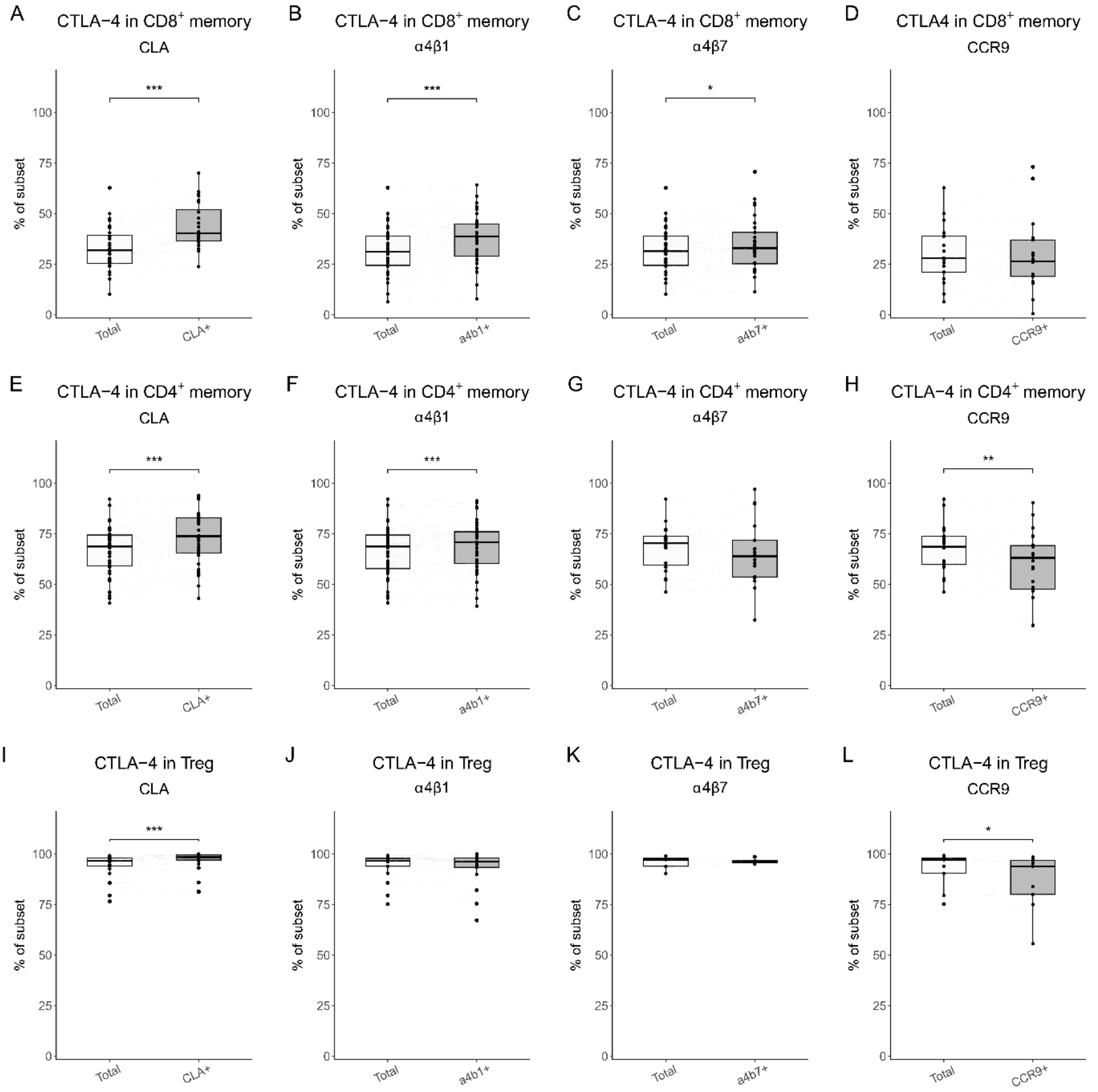

Figure S10: CTLA-4 expression in breastmilk T cells expressing homing receptors compared with total T cells (FACS, n=26). Frequency of CTLA-4^+ cells within $CD8^+CD45RA^+$ **(A-D)**, $CD4^+CD45RA^+$**(E-H)** and regulatory **(I-L)** breastmilk T cells comparing the total population with the subset of cells positive for the homing receptors CLA **(A, E, I)**, α4β1 **(B, F, J)**, α4β7 **(C,G,K)** and CCR9 **(D,H,L)**. Significance stars denotate if *=p<0.05, **=p<0.01, ***=p<0.001, Testing paired difference between expression in the total T-cell population and T-cell subset using Wilcoxon rank-sum test. Lines connect datapoints from the same breastmilk donor. Only samples with more than 50 cells within the homing receptor-positive populations were included.

Supplementary Table S2

Homing/Treg - 1 (figures: 1C; 4B-C; 5C; 6E-G; S1A; S2K; S5D,H-I; S6E-G; S9; S10)

Laser	Channel	Fluorochrome	Antibody		Clone	Company
488	Blue 1	FITC	Ki67	Intracellular	Mip1	Agilent
	Blue 3	PerCP-Cy5.5	Integrin B7	Surface	FIB27	Biolegend
640	Red 1	APC	CCR9	Surface	LO53E8	Biolegend
	Red 1/1	AF700	Integrin B1	Surface	TS2/16	Biolegend
	Red 2	APC-Cy7	CD8	Surface	SK1	BD
405	Violet 1	PB	CLA	Surface	HECA-452	Biolegend
	Violet 2	BV510	TCRgd	Surface	B1	Biolegend
	Violet 3	BV605	CD3	Surface	UCHT1	Biolegend
	Violet 4					Biolegend
	Violet 5	BV711	CD45RA	Surface	HI100	Biolegend
	Violet 6	BV785	CD4	Surface	OKT4	Biolegend
561	Y/Gr 1	PE	CTLA4	Intracellular	BNI3	BD
	Y/Gr 2	PE-Cy594	FOXP3	Intracellular	259D/C7	BD
	Y/Gr 3					
	Y/Gr 4					
	Y/Gr 5	PE-Cy7	Integrin A4	Surface	9F10	Biolegend

Cytokines (PMA/ionomycin stimulated) (figures: 4A; S4C, E-G)

Laser	Channel	Fluorochrome	Antibody		Clone	Company
488	Blue 1	FITC	GZMB	Intracellular	GB11	BD
	Blue 3	PerCP-Cy5.5	IL13	Intracellular	JES10-5A2	Biolegend
640	Red 1	AF647	TNFa	Intracellular	MAB11	Biolegend
	Red 1/1					
	Red 2	APC-Cy7	CD8	Surface	SK1	BD
405	Violet 1	PB	CLA	Surface	HECA-452	Biolegend
	Violet 2	eFluor506	Live/dead			eBioscience
	Violet 3	BV605	CD3	Surface	UCHT1	Biolegend
	Violet 4					
	Violet 5	BV711	CD45RA	Surface	HI100	Biolegend
	Violet 6	BV785	CD4	Surface	OKT4	Biolegend
561	Y/Gr 1	PE	CD69	Surface	FN50	BD
	Y/Gr 2					
	Y/Gr 3					
	Y/Gr 4					
	Y/Gr 5	PE-Cy7	IFNg	Intracellular	4S.B3	BD

CD8/T-RM (figures: 3B-D; 6A-B, D; S4A-B, S6C-D)

Laser	Channel	Fluorochrome	Antibody		Clone	Company
488	Blue 1	FITC	CD11a	Surface	Clone 38	Biorad
	Blue 3	PerCP-Cy5.5	Granzyme K	Intracellular	GM26E7	Biolegend
640	Red 1	APC	KLRD1	Surface	HP-3D9	BD
	Red 1/1	AF700	CD3	Surface	UCHT1	Biolegend
	Red 2	APC-eF780	EOMES	Intracellular	WD1928	eBioscience
405	Violet 1	PB	Granzyme B	Intracellular	GB11	BD
	Violet 2	eFluor506	Dead cell stain			eBioscience
	Violet 3	BV605	CD103	Surface	Ber-ACT8	Biolegend
	Violet 4	BV650	CD8	Surface	RPA-T8	BD
	Violet 5	BV711	CD45RA	Surface	HI100	Biolegend
	Violet 6	BV785	CD4	Surface	OKT4	Biolegend
561	Y/Gr 1	PE	CCL5	Intracellular	VL1	Biolegend
	Y/Gr 2	PE-Dazzle594	CXCR6	Surface	K041E5	Biolegend
	Y/Gr 3					
	Y/Gr 4					
	Y/Gr 5	PE-Cy7	CD69	Surface	FN50	BD

T cell subsets (figures: 1D-E; S1B-D; S2A-I)

Laser	Channel	Fluorochrome	Antibody		Clone	Company
488	Blue 1	FITC	TCRgd	Surface	IMMU510	Beckman-Coultier
	Blue 3	PerCP-Cy5.5	CD8	Surface	RPA-T8	Biolegend
640	Red 1					
	Red 1/1	AF700	CD3	Surface	UCHT1	Biolegend
	Red 2	APC-eF780	CD27	Surface	O323	eBioscience
405	Violet 1					
	Violet 2	eFluor506	Live/dead			eBioscience
	Violet 3	BV605	CD31	Surface	WM59	BD
	Violet 4					
	Violet 5	BV711	CD45RA	Surface	HI100	Biolegend
	Violet 6	BV785	CD4	Surface	OKT4	Biolegend
561	Y/Gr 1					
	Y/Gr 2					
	Y/Gr 3	PE-Cy5	CD161	Surface	DX12	BD
	Y/Gr 4					
	Y/Gr 5	PE-Cy7	TCR Vα7.2	Surface	3C10	Biolegend

Homing/Treg - 2 (figures: 5B,D; S2J; S6A-B)

Laser	Channel	Fluorochrome	Antibody		Clone	Company
488	Blue 1	FITC	CD103	Surface	2G5	Beckman-Coultier
	Blue 3	PerCP-Cy5.5	PD-1	Surface	EH12.2H7	Biolegend
640	Red 1	APC	CCR7	Surface	G043H7	Biolegend
	Red 1/1					
	Red 2	APC-Cy7	CD8	Surface	SK1	BD
405	Violet 1	eF450	FOXP3	Intracellular	PCH101	eBioscience
	Violet 2	BV510	CD3	Surface	OKT3	Biolegend
	Violet 3	BV605	CXCR3	Surface	G025H7	Biolegend
	Violet 4					
	Violet 5	BV711	CD25	Surface	2A3	BD
	Violet 6					
561	Y/Gr 1	PE	CCR5	Surface	eBioT21/8	eBioscience
	Y/Gr 2	PE-Cy594	CXCR4	Surface	12G5	BD
	Y/Gr 3	PE-Cy5	CD4	Surface	RPA-T4	BD
	Y/Gr 4					
	Y/Gr 5	PE-Cy7	CD69	Surface	FN50	BD

Sort staining (scRNAseq)

Laser	Channel	Fluorochrome	Antibody		Clone	Company
488	Blue 1	FITC	CD103	Surface	2G5	Beckman-Coultier
	Blue 3	PerCP-Cy5.5	CD25	Surface	BC96	Sony Biotechnology
640	Red 1	AF647	CD45RO	Surface	UCHL1	Biolegend
	Red 1/1		CD3	Surface	UCHT1	Biolegend
	Red 2	APC-Cy7	CD8a	Surface	SK1	BD
405	Violet 1	Sytox blue	Dead cell stain			Invitrogen
	Violet 2	BV510	TCRgd	Surface	B1	Biolegend
	Violet 3	BV605	CD127	Surface	A019D5	Sony Biotechnology
	Violet 4					
	Violet 5	BV711				
	Violet 6		CD4	Surface	OKT4	Biolegend
561	Y/Gr 1	PE	CD69	Surface	FN50	BD
	Y/Gr 2	PE-Cy594				
	Y/Gr 3	PE-Cy5				
	Y/Gr 4					
	Y/Gr 5	PE-Cy7	TCRVa7.2	Surface	3C10	Biolegend

Supplemental Acknowledgements

The following respondents of the PRIMA Initiative Group (in alphabetic order) opted to have their name acknowledged :

- André C. Knulst; Department of Dermatology/Allergology, University Medical Center Utrecht, Utrecht, The Netherlands.

- Bernd Stahl; Division of Pharmacology, Faculty of Science, Utrecht Institute for Pharmaceutical Sciences, Utrecht University, Utrecht, Netherlands. Global Centre of Excellence Immunology, Danone Nutricia Research B.V., Utrecht, Netherlands. Global Centre of Excellence Human Milk Research and Analytical Sciences, Danone Nutricia Research B.V., Utrecht, Netherlands.

- C. Kors van der Ent; Department of Pediatric Pulmonology and Allergology, Wilhelmina Children's Hospital/University Medical Center, Utrecht University, Utrecht, the Netherlands.

- Caroline G.M. de Theije; Department of Neonatology, Wilhelmina Children's Hospital/University Medical Centre Utrecht, Utrecht, The Netherlands.

- Debbie van Baarle; Center for Translational Immunology, University Medical Center Utrecht, Utrecht, Netherlands. Centre for Infectious Disease Control, National Institute for Public Health and the Environment (RIVM) , Bilthoven, The Netherlands.

- Elisabeth A.M. Sanders; Center for Translational Immunology, University Medical Center Utrecht, Utrecht, Netherlands. Centre for Infectious Disease Control, National Institute for Public Health and the Environment (RIVM) , Bilthoven, The Netherlands.

- Gaby Smits; Centre for Infectious Disease Control, National Institute for Public Health and the Environment (RIVM), Bilthoven, The Netherlands.

- Gerco den Hartog; Centre for Infectious Disease Control, National Institute for Public Health and the Environment (RIVM) , Bilthoven, The Netherlands.

- Hanneke Linde; Center for Translational Immunology, University Medical Center Utrecht, Utrecht, Netherlands

- Ilse van den Bosch-Spangler; Department of Obstetrics and Gynaecology, Diakonessenhuis, Utrecht, the Netherlands.

- Johan Garssen; Head Division Pharmacology, Utrecht Institute for Pharmaceutical Sciences, Utrecht University, Utrecht, The Netherlands. Danone Nutricia Research, Utrecht, Netherlands.

- Kitty W.M. Bloemenkamp; Department of Gynaecology and Obstetrics, University Medical Center Utrecht, Utrecht, the Netherlands.

- Koen L. Deurloo ; Department of Obstetrics and Gynaecology, Diakonessenhuis, Utrecht, the Netherlands.

- Laura A.P.M. Meulenbroek; Global Centre of Excellence Immunology, Danone Nutricia Research B.V., Utrecht, Netherlands.

- Léon M.J. Knippels; Friesland-Campina, Amersfoort, The Netherlands.

- Lilly M. Verhagen; Department of Paediatric Immunology and Infectious Diseases, Wilhelmina Children's Hospital/University Medical Center Utrecht, Utrecht, Netherlands. Department of Pediatric Infectious Diseases and Immunology, Amalia Children's Hospital, Radboud Center for Infectious Diseases, Radboud University Medical Center, Nijmegen, The Netherlands. Laboratory of Medical Immunology, Radboud Institute for Molecular Life Sciences, Radboud University Medical Center, Nijmegen, The Netherlands

- Lisa A. van den Berg; Center for Translational Immunology, University Medical Center Utrecht, Utrecht, Netherlands

- Maaike Nederend; Center for Translational Immunology, University Medical Center Utrecht, Utrecht, Netherlands

- Marca H.M. Wauben; Department of Biomolecular Health Sciences, Faculty of Veterinary Medicine, Utrecht University, Utrecht, the Netherlands.

- Marjan Kuijer; Centre for Infectious Disease Control, National Institute for Public Health and the Environment (RIVM), Bilthoven, The Netherlands.

- Martijn J.C. van Herwijnen; Department of Biomolecular Health Sciences, Faculty of Veterinary Medicine, Utrecht University, Utrecht, the Netherlands.

- Nynke Y. Rots, Center for Translational Immunology, University Medical Center Utrecht, Utrecht, Netherlands. Centre for Infectious Disease Control, National Institute for Public Health and the Environment (RIVM) , Bilthoven, The Netherlands.

- Sabine M.P.J. Prevaes; Department of Pediatric Pulmonology and Allergology, Wilhelmina Children's Hospital/University Medical Center, Utrecht University, Utrecht, the Netherlands.

- Tanja A.M. Voogt-Vrijhoef; Department of Obstetrics and Gynaecology, Diakonessenhuis, Utrecht, the Netherlands.

- Thuy-Me Le; Department of Dermatology/Allergology, University Medical Center Utrecht, Utrecht, The Netherlands.

- Wouter J. de Waal (MD, PhD); Department of Pediatrics, Diakonessenhuis, Utrecht, The Netherlands.
